# cAMP-dependent regulation of HCN4 controls the tonic entrainment process in sinoatrial node pacemaker cells

**DOI:** 10.1038/s41467-020-19304-9

**Published:** 2020-11-03

**Authors:** Stefanie Fenske, Konstantin Hennis, René D. Rötzer, Verena F. Brox, Elvir Becirovic, Andreas Scharr, Christian Gruner, Tilman Ziegler, Verena Mehlfeld, Jaclyn Brennan, Igor R. Efimov, Audrys G. Pauža, Markus Moser, Carsten T. Wotjak, Christian Kupatt, Rasmus Gönner, Rai Zhang, Henggui Zhang, Xiangang Zong, Martin Biel, Christian Wahl-Schott

**Affiliations:** 1 Center for Integrated Protein Science (CIPS-M) and Center for Drug Research, Department of Pharmacy, Ludwig-Maximilians-Universität München, 81377 Munich, Germany; 2 German Center for Cardiovascular Research (DZHK), Partner Site Munich Heart Alliance, 80802 Munich, Germany; 3 Klinikum Rechts der Isar, TU Munich, 81675 Munich, Germany; 4 Department of Biomedical Engineering, George Washington University, Washington, DC 20052 USA; 5 Institute of Anatomy, Lithuanian University of Health Sciences, LT, 44307 Kaunas, Lithuania; 6 Max Planck Institute of Biochemistry, Department of Molecular Medicine, 82152 Martinsried, Germany; 7 Max Planck Institute of Psychiatry, Research Group Neuronal Plasticity, 80804 Munich, Germany; 8 Central Nervous System Diseases Research, Boehringer Ingelheim Pharma GmbH & Co. KG, 88397 Biberach Riß, Germany; 9 Department of Aerospace Engineering, University of Bristol, Bristol, BS8 1QU UK; 10 Department of Physics and Astronomy, The University of Manchester, Manchester, M13 9PL UK; 11 Hannover Medical School, Institute for Neurophysiology, 30625 Hannover, Germany

**Keywords:** Cyclic nucleotide-gated cation channels, Autonomic nervous system, Cardiology, Arrhythmias

## Abstract

It is highly debated how cyclic adenosine monophosphate-dependent regulation (CDR) of the major pacemaker channel HCN4 in the sinoatrial node (SAN) is involved in heart rate regulation by the autonomic nervous system. We addressed this question using a knockin mouse line expressing cyclic adenosine monophosphate-insensitive HCN4 channels. This mouse line displayed a complex cardiac phenotype characterized by sinus dysrhythmia, severe sinus bradycardia, sinus pauses and chronotropic incompetence. Furthermore, the absence of CDR leads to inappropriately enhanced heart rate responses of the SAN to vagal nerve activity in vivo. The mechanism underlying these symptoms can be explained by the presence of nonfiring pacemaker cells. We provide evidence that a tonic and mutual interaction process (tonic entrainment) between firing and nonfiring cells slows down the overall rhythm of the SAN. Most importantly, we show that the proportion of firing cells can be increased by CDR of HCN4 to efficiently oppose enhanced responses to vagal activity. In conclusion, we provide evidence for a novel role of CDR of HCN4 for the central pacemaker process in the sinoatrial node.

## Abstract (web summary)

The involvement of cAMP-dependent regulation of HCN4 in the chronotropic heart rate response is a matter of debate. Here the authors use a knockin mouse model expressing cAMP-insensitive HCN4 channels to discover an inhibitory nonfiring cell pool in the sinoatrial node and a tonic and mutual interaction between firing and nonfiring pacemaker cells that is controlled by cAMP-dependent regulation of HCN4, with implications in chronotropic heart rate responses.

## Introduction

Throughout life, the human heart beats up to 3 billion times with high precision and without rest. The heartbeat is initiated in the leading pacemaker region within the sinoatrial node. This region is a small cluster of pacemaker cells that display a faster firing rate than the residual cells in the SAN and cardiac conduction system. Within this region individual pacemaker cells synchronize to a common rhythm by mutual, electrical interaction via gap junctions. This mechanism is called mutual entrainment^1–4^ and is important for the ability of the leading pacemaker region to generate regular electrical discharges that drive the electrical activation of the whole heart. The assumption that spontaneous activity of the leading pacemaker region of the SAN is characterized by the fastest firing rate and that this drives the heart rate (HR) represents the central premise of the pacemaker process. Spontaneous firing of pacemaker cells is initiated by the slow diastolic depolarisation^5^. Hyperpolarization-activated cyclic nucleotide-gated cation (HCN) channels are considered essential for the slow diastolic depolarisation^6^. The four HCN1–4 subtypes are principally operated by hyperpolarisation; however, activation is also controlled by cyclic adenosine monophosphate (cAMP). It is known that cAMP binds to a cyclic nucleotide-binding domain in the C-terminus of the channels^7^. Within the physiological voltage range, HCN channel activity increases in parallel with intracellular cAMP concentrations^8^. Moreover, it has been proposed that CDR of HCN4 is key for heart rate control by the autonomic nervous system (ANS). Specifically, cAMP-dependent increase of HCN4-mediated current was believed to be required for the acceleration of the HR upon high activity of the sympathetic division of the ANS, whereas a drop in cAMP levels following vagal stimulation would decrease HCN4 activity and slow down the HR^5,6,9^.

While the assumption, that CDR of HCN4 underlies HR control by the ANS has been postulated for quite a long time, it has been difficult to validate this concept in vivo and it is therefore still a matter of controversy. In particular, there are conflicting results and conclusions about a potential role of cAMP binding to the cyclic nucleotide-binding domain of HCN4 channels during the chronotropic response. While experiments on embryonic mouse hearts expressing a mutant HCN4 channel lacking CDR indicated that CDR of HCN4 is crucial for HR control^10^, results of tamoxifen-induced knockout of HCN4 in adult mouse hearts argued against a major role for HCN4 in HR control^11,12^. Moreover, several HCN4 channelopathies in humans (congenital diseases caused by mutations in genes coding for ion channels) lead to heterogeneous cardiac syndromes including bradycardia, yet in some of these channelopathies the response to β-adrenergic stimulation is preserved^13,14^. Unfortunately, the only two known human mutations that directly impair cAMP-modulation (573X and 695X) lead to large truncations of the HCN4 C-terminus and affect general HCN4 architecture^15,16^. Therefore, a straight-forward interpretation of the impact of these mutations on CDR is not possible. Moreover, all patients with HCN4 channelopathies identified so far are heterozygous for the respective mutation and thus also express a “healthy” copy of the HCN4 channel.

Recently, alternative concepts emerged indicating that HCN channels might have an entirely different role for HR regulation than originally postulated. There is evidence that mechanisms are required, which oppose and control HR responses to sympathetic or parasympathetic activity during the chronotropic response, ensuring balanced and well-tuned HR changes. Importantly, HCN channels, together with other signalling proteins^17–19^, could play a major role within this context and represent key targets, which control and oppose the HR-lowering effect of the parasympathetic nervous system and thereby prevent parasympathetic override, inappropriate HR decreases and severe bradycardia^1,20–22^.

In order to directly investigate the role of HCN4 CDR in SAN function we generated a knockin mouse line expressing cAMP-insensitive HCN4 channels. In vivo and in vitro characterization of this mouse line revealed a complex cardiac phenotype characterized by severe sinus bradycardia and sinus dysrhythmia, but fully preserved HR regulation. Furthermore, the absence of CDR leads to inappropriately enhanced HR responses of the SAN to vagal nerve activity during the baroreceptor reflex in vivo. Moreover, arrhythmia arises in the absence of HCN4 CDR during high adrenergic activity because subsidiary pacemaker regions take over pacemaker function in place of the SAN. Finally, we elaborate and propose a model based on cellular, organ and in vivo experiments which explains the cellular mechanism underlying this complex cardiac phenotype. Together, our study provides evidence for a novel role of CDR of HCN4 for pacemaking in the SAN and in particular for the central pacemaker process.

## Results

### Generation and validation of HCN4FEA mice

**Fig. 1:**
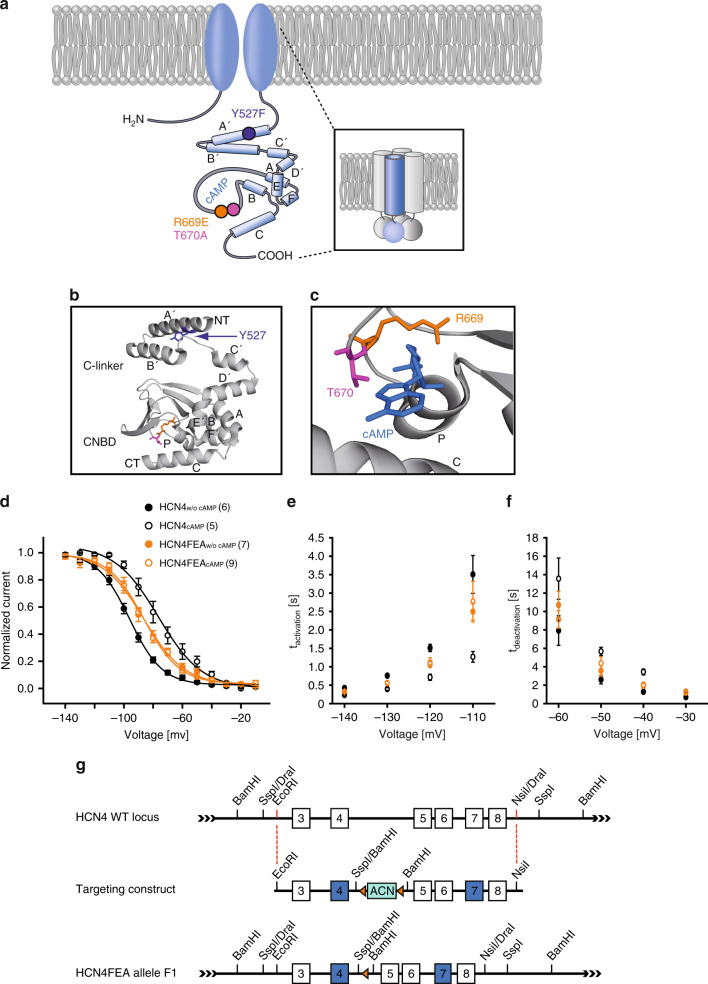
Generation of HCN4FEA mice and basic characterisation of HCN4FEA-mediated I_f_. **a** Illustration depicting the HCN4 channel tetramer (inset) and single subunit. The C-terminus contains the C-linker composed of six α-helices (A’–F’) and a cyclic nucleotide-binding domain (autonomic) composed of three α-helices (A–C) and a β-roll between helix A and B. Localisation of the three introduced point mutations is indicated with coloured circles. **b** C-terminus of the human HCN4 channel (PDB #Q9Y3Q4). Residues Y527 (purple), R669 (orange), and T670 (pink) are highlighted. The R669E and T670A mutations were introduced to completely disrupt cAMP binding. Both residues are located in the loop between the P helix and the β7 sheet of the CNBD and are highly conserved throughout the CNG (cyclic nucleotide-gated) and HCN channel family^7,53^. **c** cAMP bound to the CNBD of human HCN4. Residues R669 and T670 are required for cAMP binding. **d** Activation curves of WT HCN4 and HCN4FEA channels transiently expressed in HEK293 cells in the absence and presence of 100 µM cAMP in the pipette solution. A holding potential of −40 mV was used. cAMP shifts the activation curve of WT HCN4 to more depolarised potentials, whereas HCN4FEA activation curves are not affected by cAMP. **e** Activation time constants determined from HCN4 and HCN4FEA-mediated currents measured at test potentials ranging from −140 mV to −110 mV. Values from HCN4FEA mutant channels lie between values determined from WT channels with and without cAMP. **f** Deactivation time constants determined from currents measured at test potentials ranging from −60 mV to −30 mV. Values from HCN4FEA mutant channels lie between values determined from WT channels with and without cAMP. **g** Targeting strategy of HCN4FEA knockin animals. The HCN4 WT locus comprises exons 3–8. Exons depicted in blue carry the mutations. In the targeting construct, two substitutions were located in the CNBD (R669E, T670A) and one in the C-linker (Y527F) of the channel protein. Residues R669 and T670 are located in the loop between the P helix and the β7 sheet of the CNBD. Both residues directly interact with the phosphate group of cAMP. Data represent the mean ± SEM, n numbers are given in parentheses. Source data are provided as a Source Data file.

To examine the role of HCN4 CDR in the heart, we generated HCN4FEA knockin mice that contain three amino acid exchanges in HCN4 (Y527F, R669E, and T670A; Fig. 1). The R669E and T670A mutations disrupt cAMP binding and completely abolish channel activation by cyclic nucleotides (Supplementary Data 1a, b)^7,10,23^. Previous studies indicated that abolishing the cAMP binding capability of the HCN4 cyclic nucleotide-binding domain is associated with embryonic lethality^10,24^. This finding possibly reflects the fact that the activation thresholds of these HCN4 mutants are more negative than the maximum diastolic potential of pacemaker cells, which is essentially as if no cAMP was present in the cell. This causes an almost complete loss of HCN4 activity at physiological voltages, which is similar to knocking out HCN4^12^.

It has been shown that in SAN cells, basal intracellular cAMP concentrations are never zero^25,26^, and that basal cAMP increases HCN4-mediated currents at any given membrane potential in the physiological voltage range. To account for this scenario, we introduced a third mutation in the C-linker of the channel (Y527F). This mutation causes a parallel shift of the activation curve to more positive potentials and acceleration of activation kinetics in the same range as expected in WT channels under basal cAMP levels, i.e. *V*_0.5_ and activation time constants values are in between those obtained for WT channels measured in the absence and in the presence of cAMP (Supplementary Data 1as).

The HCN4Y527F channels combined with R669E/T670A display (1) a constant and cAMP-independent shift of the half-maximal activation voltage (*V*_0.5_) towards more depolarised potentials and (2) an acceleration of activation kinetics. These parameters were in between respective parameters obtained for wild-type channels in the absence and presence of cAMP. Channel properties were not modulated by cAMP-even at high micromolar concentrations (Fig. 1d–f and Supplementary Data 1a, b).

**Fig. 2:**
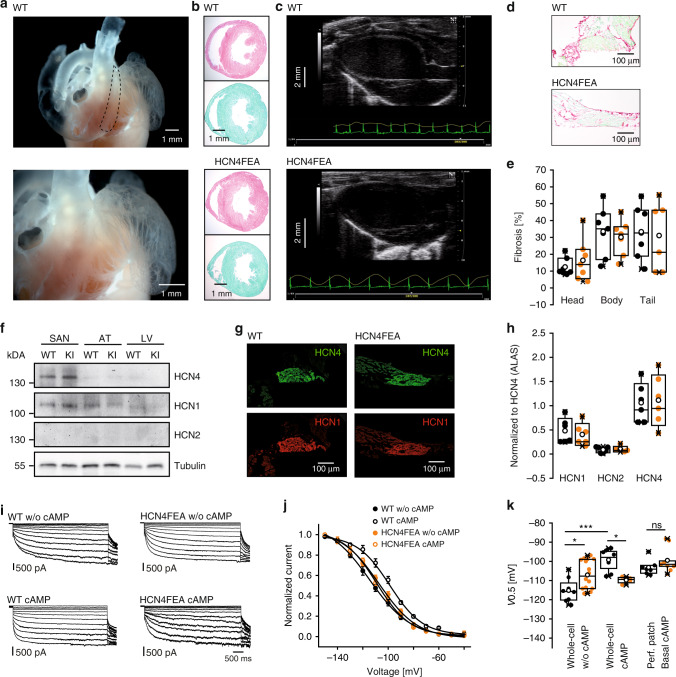
Similar expression and distribution of HCN channels in the WT and HCN4FEA heart. **a** Gelatin-inflated heart preparation depicting the anatomy of the SAN region (*n* = 5 biologically independent samples). **b** Hematoxylin and eosin (H&E, *n* = 6 WT + 6 HCN4FEA biologically independent samples) and fibrosis stainings (Sirius red/ Fast green, *n* = 7 WT + 7 HCN4FEA biologically independent samples) of ventricular cross sections. **c** Representative long-axis echocardiograms (*n* = 12 WT + 11 HCN4FEA animals). **d** Sirius red/Fast green stained transverse sections of the central SAN (SAN head, *n* = 7 WT + 7 HCN4FEA biologically independent samples). **e** Quantification of fibrosis in the SAN head (*n* = 7 WT + 7 HCN4FEA biologically independent samples), body (*n* = 7 WT + 7 HCN4FEA biologically independent samples), and tail (*n* = 7 WT + 6 HCN4FEA biologically independent samples). **f** HCN channel expression in SAN, atria (AT), and left ventricle (LV) of WT and HCN4FEA (KI) mice (one blot with pooled tissue from 6 WT + 6 HCN4FEA animals). Tubulin: loading control. **g** Consecutive sections of the central SAN shown in **d** demonstrating the transmural distribution of HCN4 (green) and HCN1 (red) in WT and HCN4FEA (*n* = 3 WT + 3 HCN4FEA biologically independent samples). **h** Quantitative PCR analysis of HCN channel transcript levels from SANs normalised to HCN4 channel expression (*n* = 6 WT + 6 HCN4FEA biologically independent samples). Expression levels of HCN1 and HCN2 were also comparable between both genotypes. **i** Representative whole-cell I_f_ recordings from isolated SAN cells under conditions without (upper panel) and with (lower panel) 100 µM cAMP in the pipette solution. **j** Averaged I_f_ activation curves determined from current measurements as shown in **i** (WT w/o cAMP *n* = 8 cells, WT cAMP *n* = 8 cells, HCN4FEA w/o cAMP *n* = 16 cells, HCN4FEA cAMP *n* = 4 cells; data are presented as mean values ±:SEM). **k** Half-maximal activation voltage determined from I_f_ activation curves in whole-cell configuration without (*n* = 8 WT + 16 HCN4FEA cells) and with 100 µM cAMP in the pipette solution (*n* = 8 WT + 4 HCN4FEA cells) and from perforated-patch configuration with basal cAMP levels (*n* = 7 WT + 9 HCN4FEA cells). WT w/o cAMP vs HCN4FEA w/o cAMP: *p* = 0.0075; WT w/o cAMP vs WT cAMP: *p* = 0.00003; WT cAMP vs HCN4FEA cAMP: *p* = 0.0127; WT basal cAMP vs HCN4FEA basal cAMP (perforated-patch): *p* = 0.2126. All experiments were performed using male mice. Boxplots show the median line, perc 25/75, and min/max value; open symbols represent the mean value. Significance levels: *two-way ANOVA. Source data are provided as a Source Data file.

Heterozygous breeding produced offspring at the expected Mendelian ratios. Homozygous HCN4FEA mice did not show any morphological abnormalities and body weights were similar to those of WT littermates (Supplementary Data 2a). Overall cardiac morphology appeared normal, histological sections of WT and HCN4FEA hearts were indistinguishable (Fig. 2a, b), and echocardiographic analysis demonstrated normal cardiac structure as well as diastolic and systolic function in both groups of mice (Fig. 2c, Supplementary Data 2b). Detailed analysis of SAN cross sections revealed no evidence of fibrosis or myofibrillar disarray (Fig. 2d, e, Supplementary Data 2c).

### Expression of HCN channels in the heart

Quantitative reverse-transcription polymerase chain reaction confirmed that HCN4 WT and HCN4FEA are expressed at similar levels in the SAN of WT and HCN4FEA mice, respectively (Fig. 2h, Supplementary Data 2d). This finding is supported by a western blot analysis of the membrane fractions of SAN preparations (Fig. 2f), which indicated similar levels of a 135-kDa signal corresponding to the mature glycosylated HCN4 or HCN4FEA protein that was present in the SAN of WT and HCN4FEA mice. Protein distribution of HCN4 and HCN1 channels in the head, body and tail region of the SAN and surrounding atrial tissue were similar (Fig. 2f, g; Supplementary Data 2e). Gene expression profiles of isolated SAN tissue (Supplementary Data 3) confirmed that the expression levels of major depolarising and repolarising ion channels or proteins contributing to SAN action potentials were not altered in HCN4FEA mice. Together, the results indicate that compensatory remodelling or changes in gene expression profile were not relevant issues in the HCN4FEA heart.

### Native I_f_ properties in isolated SAN pacemaker cells

In single pacemaker cells isolated from the SAN, hyperpolarising voltage steps activated robust I_f_ (Fig. 2i). Steady-state I_f_ current densities of WT and HCN4FEA cells, determined at −140 mV in whole-cell mode, were similar in the absence of exogenous cAMP and in presence of 100 µM cAMP in the intracellular recording solution (Supplementary Data 1b). The slow (τau1) and fast (τau2) activation time constants representing activation of HCN4 and HCN1 channels, were similar in WT and HCN4FEA cells (Supplementary Data 1b) in the absence of cAMP. In contrast, intracellular cAMP accelerated the activation kinetics of WT, but not HCN4FEA channels. Steady-state activation curves were determined to derive the half-maximal activation voltage (*V*_0.5_) and the slope values (*k*) (Fig. 2j, k, Supplementary Data 1b). In the absence of cAMP, *V*_0.5_ values in HCN4FEA cells were more positive than WT values, which is in agreement with data from heterologously expressed HCN4FEA channels (Fig. 1d and Supplementary Data 1a). Cyclic AMP induced a significant right shift of the *V*_0.5_ value of WT but not HCN4FEA activation curves. Slope factors in the absence and presence of cAMP were similar between WT and HCN4FEA cells. In perforated-patch measurements where endogenous cAMP concentrations are present^27^, *V*_0.5_ values were similar in WT and HCN4FEA cells (Fig. 2k, Supplementary Data 1c).

### Sinus node bradycardia in HCN4FEA mice

**Fig. 3:**
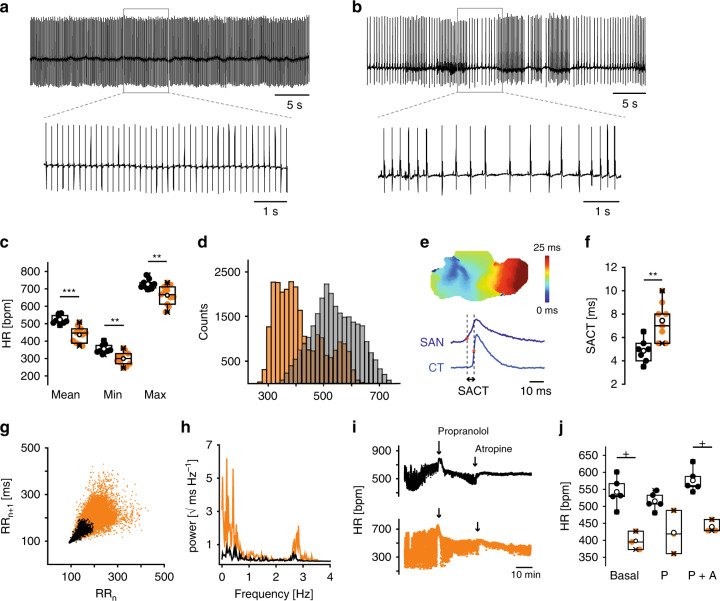
HCN4FEA mice display bradycardia and sinus dysrhythmia. **a,** **b** Telemetric ECG traces obtained from a conscious, freely moving **a** WT mouse and **b** HCN4FEA mouse. Insets: Extended ECG traces. **c** Mean, minimum, and maximum HR determined from 72-h RR intervals (WT: *n* = 9, HCN4FEA: *n* = 11; two-sided *t*-test; mean HR: *p* = 0.0019, min HR: *p* = 0.0040, max HR: *p* = 0.0383). **d** HR histograms of a WT (light gray) and HCN4FEA mouse (orange) determined from 72-h (12 h/12 h light/dark cycle) ECGs. **e** Determination of sinoatrial conduction time) with optical imaging measurements. Activation map of a biatrial WT SAN preparation (upper panel) and optical action potentials (OAP) (lower panel) from the leading pacemaker site in the SAN (dark blue) and from the earliest atrial excitation site within the crista terminalis (CT; light blue). SACT is determined as the time difference of activation time points indicated by asterisk. **f** SACT in WT and HCN4FEA SAN preparations (*n* = 7 WT + 8 HCN4FEA biologically independent samples; two-sided *t*-test; *p* = 0.0029). **g** Comet-shaped Poincaré plots in HCN4FEA mice (orange). **h** Frequency domain power spectral density plots obtained from a WT (black) and HCN4FEA (orange) mouse. **i** Tachograms of WT (black) and HCN4FEA (orange) mice before and after subsequent injection of propranolol (20 mg/kg i.p) and atropine (1 mg/kg i.p). Arrows indicate timepoint of injection (WT: *n* = 5, HCN4FEA: *n* = 3). **j** Mean basal HR and HR after successive injection of propranolol and atropine (*n* = 5 WT + 3 HCN4FEA animals; Mann–Whitney U test; basal: *p* = 0.03571, P: *p* = 0.07143, P + A: *p* = 0.03571). In vivo experiments were performed using male animals. In vitro experiments were performed using tissue isolated from female animals. Boxplots show the median line, perc 25/75, and min/max value; open symbols represent the mean value. Significance levels: *student´s paired *t*-test; ^+^Mann–Whitney U test. Source data are provided as a Source Data file.

Telemetric ECG recordings^1,20^ (Fig. 3a–d, Supplementary Data 4a) revealed that the average and minimum HRs were significantly decreased in HCN4FEA mice indicating pronounced bradycardia. In addition, HCN4FEA animals were not able to increase their maximum HR to the same level as their WT littermates. This condition is clinically defined as chronotropic incompetence. Strikingly, HR regulation dynamics (HR_max_/HR_min_) and HR range (HR_max_-HR_min_) were preserved in the HCN4FEA mouse (Supplementary Data 4a–b). Seventy-two-hour HR histograms were shifted toward lower values in the HCN4FEA animals (Fig. 3d). Furthermore, WT histograms of HR were symmetrical with a centred peak. In contrast, histograms of HCN4FEA showed left-skewed distribution with a peak in the low HR range. In-line with the manifest bradycardia observed in vivo, markedly reduced beating rates were also present in vitro in preparations containing the intact sinoatrial node network with surrounding tissue (isolated whole hearts and biatrial preparations) of the HCN4FEA mouse (Supplementary Data 6a–b, 8a).

To further investigate the integrity of sinus node automaticity and conduction in vivo an intracardiac electrophysiological study was performed (Supplementary Fig. 1). This revealed an increase in sinus node recovery time, which is defined as the time required to reinitiate spontaneous firing following overdrive pacing. This indicated a delayed impulse formation within the HCN4FEA SAN. In addition, premature atrial stimulation^20^ (Supplementary Fig. 1b, c and Supplementary Data 5a) as well as optical imaging (Fig. 3e, f and Supplementary Data 6d) uncovered prolonged sinoatrial conduction time in HCN4FEA SAN, yet the conduction pathway throughout the SAN and atria was similar to WT (Supplementary Fig. 2a–d). The combination of impaired impulse formation and conduction causes severe sinus bradycardia and a reduction in cardiac output. Furthermore, electrophysiological study revealed that atrioventricular (AV) node function was normal in HCN4FEA mice (Supplementary Fig. 1d–k and Supplementary Data 5a). Qualitative immunohistochemical experiments and Sirius red/Fast green stainings suggested that HCN4 protein levels and distribution, and amount of fibrous tissue in the AV node were similar to WT (Supplementary Fig. 1l, m).

### Sinus dysrhythmia in HCN4FEA mice

Besides bradycardia and chronotropic incompetence, severe sinus dysrhythmia was dominating ECG traces of HCN4FEA mice (Fig. 3b). Sinus dysrhythmia is clinically defined as irregular HR characterized by large beat to beat variations originating in the SAN. Sinus dysrhythmia was quantified by applying signal processing tools developed for the analysis of heart rate variability in the time- and frequency domain^1^. This revealed, that time domain parameters were significantly higher for HCN4FEA animals compared to the WT (Supplementary Data 4c). Likewise, Poincaré plots displayed higher beat-to-beat dispersion and a broad comet-shaped pattern (Fig. 3g). Frequency domain analysis of HR variability (HRV) revealed markedly increased power across all frequency bands for HCN4FEA compared with WT (Fig. 3h, Supplementary Data 4d). Overall, fluctuations were more pronounced during phases of slow HR. To study the effect of complete autonomic blockade on HRV parameters, atropine and propranolol were administered consecutively. Treatment of WT and HCN4FEA mice with propranolol reduced HR fluctuations, which was further reduced upon subsequent treatment with atropine (Fig. 3i, Supplementary Data 4e). This indicates that the major part of severe HR fluctuations in HCN4FEA animals are attributable to ANS inputs. However, HR fluctuations were still more pronounced in HCN4FEA after complete autonomic blockade, indicating that a second, but minor part of HR fluctuations observed in vivo is independent of the ANS and attributable to intrinsic sinus node dysfunction. In support of this conclusion, HR fluctuations in in vitro preparations (isolated perfused hearts and biatrial preparations; Supplementary Data 6a, 8a)—in which intrinsically autonomic regulation is lacking—were lower than those observed in vivo for both mouse groups but were still more pronounced in HCN4FEA preparations.

### Long-lasting periods of nonfiring in HCN4FEA SAN cells

**Fig. 4:**
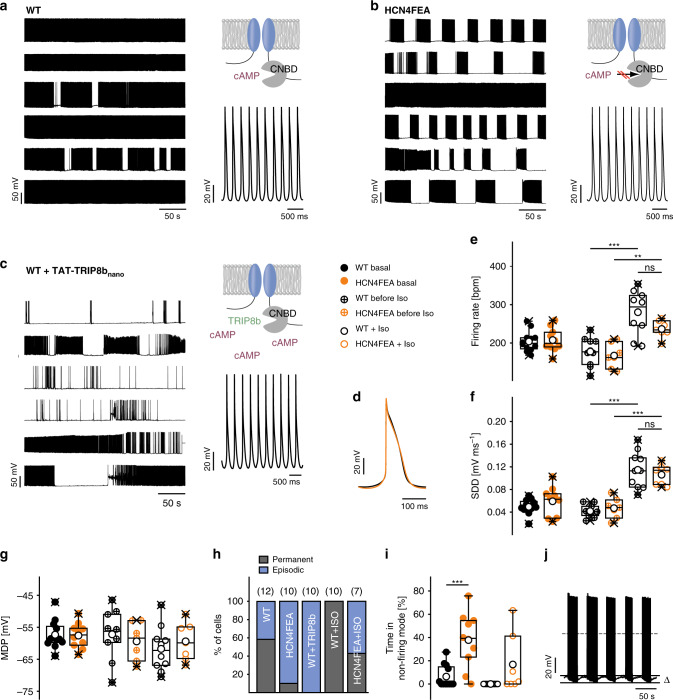
Long-lasting periods of nonfiring in isolated pacemaker cells of HCN4FEA mice. **a–c** (right, upper panel) Cartoons of channel variant used in experiments shown in the right panel. (Left panel) long-term perforated-patch-clamp recordings of spontaneous action potentials under baseline conditions in six SAN cells isolated from **a** WT and **b** HCN4FEA mice and **c** WT cells after application of TAT-TRIP8b_nano_. (Right, lower panel) Magnification of action potential recordings shown in the left panels. **d** Averaged action potentials obtained from representative perforated-patch-clamp recordings of a WT and HCN4FEA pacemaker cell. **e–g** firing rate (WT before Iso vs WT Iso: *p* = 0.000004; HCN4FEA before Iso vs HCN4FEA Iso: *p* = 0.0035; WT Iso vs HCN4FEA Iso: *p* = 0.0386), slope of slow diastolic depolarisation (SDD) (WT before Iso vs WT Iso: *p* = 0.00000003; HCN4FEA before Iso vs HCN4FEA Iso: *p* = 0.00002; WT Iso vs HCN4FEA Iso: *p* = 0.4315), and maximum diastolic potential (MDP) under basal conditions (*n* = 12 WT + 10 HCN4FEA cells) as well as before (*n* = 10 WT + 7 HCN4FEA cells) and during (*n* = 10 WT + 7 HCN4FEA cells) application of isoproterenol (100 nM). Values were determined from episodes with constant firing in WT and HCN4FEA cells. **h** Quantification of different firing patterns determined from similar recordings as shown in **a**–**c** (left panels). **i** Percentage of time spent in the nonfiring mode (basal *n* = 12 WT + 10 HCN4FEA cells, Iso *n* = 10 WT + 7 HCN4FEA cells; WT basal vs HCN4FEA basal: *p* = 0.0004). **j** Representative sequence of firing and nonfiring in an HCN4FEA pacemaker cell. The difference between MDP during firing and nonfiring was 7.14 ± 0.36 mV (Δ*V*m; *n* = 9). All experiments were performed using male animals. Boxplots show the median line, perc 25/75, and min/max value; open symbols represent the mean value. Significance levels: *Holm’s–Sidak post-hoc test following two-way ANOVA. Source data are provided as a Source Data file.

To test SAN activity at the cellular level, spontaneous firing of pacemaker cells was investigated using long-term perforated-patch-clamp experiments, which allow for stable recordings for up to 1 h (Fig. 4). Cells from either WT or HCN4FEA mice fired rhythmic, spontaneous pacemaker potentials (Fig. 4a, b, and Supplementary Data 7a) and averaged action potential shape, mean rate of spontaneous firing, slope of slow diastolic depolarisation and maximum diastolic potential were similar (Fig. 4d–g). Upon application of the β-adrenoceptor agonist isoproterenol (100 nM) AP firing rate increased in WT and HCN4FEA cells to the same extent (Fig. 4e and Supplementary Data 7e). From these findings we can conclude, that at least CDR of HCN4 and also most likely HCN4 channels in general are not involved in baseline firing of pacemaker cells.

**Fig. 5:**
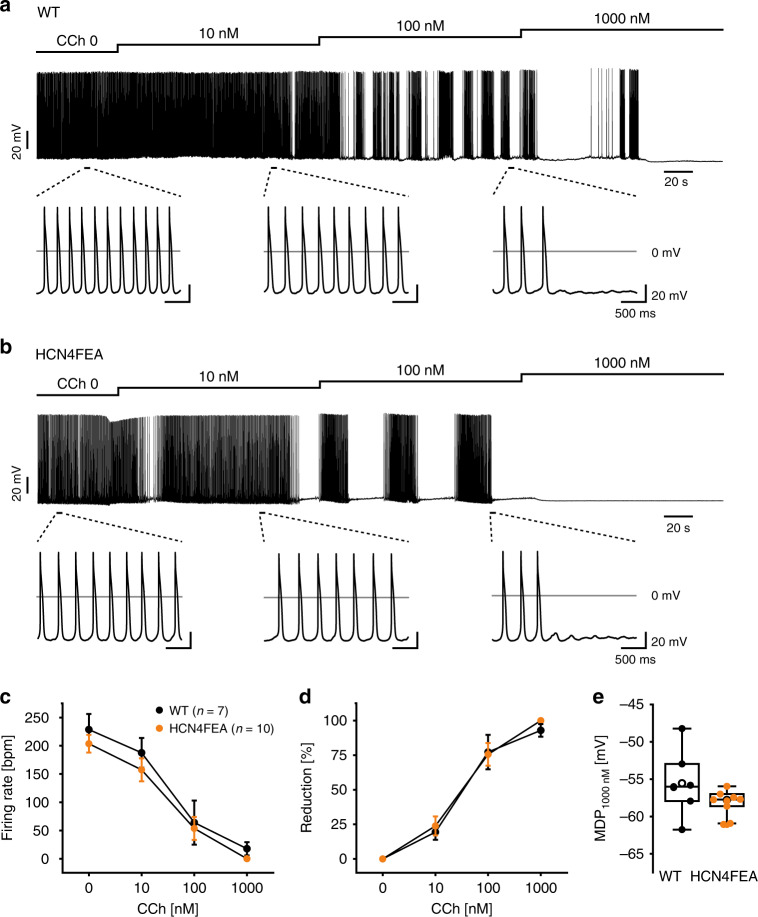
Carbachol induces episodic firing patterns with nonfiring states in both WT and HCN4FEA SAN cells. **a, b** Representative current-clamp recordings obtained in perforated-patch-clamp mode from **a** WT and **b** HCN4FEA cells. Increasing concentrations of carbachol consecutively reduced the firing frequency and caused an episodic firing pattern with nonfiring states upon successive hyperpolarisation. **c** Cumulative carbachol dose response of WT and HCN4FEA cells (*n* = 7 WT + 10 HCN4FEA cells; data are presented as mean values ± SEM). **d** Percentage reduction was calculated by normalising each value to the basal firing rate (*n* = 7 WT + 10 HCN4FEA cells; data are presented as mean values ± SEM). **e** Maximum diastolic potential after application of 1000 nM Carbachol (*n* = 7 WT + 10 HCN4FEA cells). All data were obtained from SAN cells of male WT (black) and HCN4FEA (orange) mice. Boxplots show the median line, perc 25/75, and min/max value; open symbols represent the mean value. Source data are provided as a Source Data file.

Surprisingly, in 90% of HCN4FEA cells rhythmic firing was often accompanied by a slow and progressive hyperpolarisation (HCN4FEA: Δ*V*_m_ = 7.17 ± 0.36 mV; *n* = 9; WT: Δ*V*_m_ = 8.16 ± 1.36 mV; *n* = 5; Fig. 4a, b, j), leading to extended periods of nonfiring which lasted for 28.9 ± 3.3 seconds. Subsequently, pacemaker cells slowly recovered from the hyperpolarised potential and depolarised until they restarted regular firing. This firing pattern occurred less frequently in WT cells (42%; Fig. 4h), where nonfiring episodes lasted for 16.2 ± 1.5 seconds. In total, HCN4FEA cells interrupted firing for 37.7 ± 7.4% of the total measurement time, whereas WT cells stopped firing for only 6.4 ± 2.8% of the time (Fig. 4i and Supplementary Data 7a). This finding suggests the presence of a thus far unknown, nonfiring mode of SAN cells. To test whether nonfiring can be induced by acutely switching off CDR of HCN4, we employed a plasma membrane permeable peptide (TAT-TRIP8b_nano_) derived from TRIP8b—an auxiliary subunit of neuronal HCN channels^28^. TAT-TRIP8b_nano_ was shown to bind to the cyclic nucleotide-binding domain of HCN channels, inhibiting CDR. Application of the peptide induced the nonfiring mode in WT cells followed by firing recovery (Fig. 4c); these responses were more pronounced than in HCN4FEA cells. To mimic vagal activity and to test whether a reduction in cAMP levels can induce the nonfiring state, we applied carbachol to WT and HCN4FEA cells. Indeed, carbachol induced the nonfiring state in single pacemaker cells of WT and HCN4FEA cells (Fig. 5a–d and Supplementary Data 7b). To test the effect of increased cAMP levels we applied isoproterenol to the cells. This completely abolished the nonfiring mode in WT cells and reduced the amount of HCN4FEA cells that displayed the nonfiring mode (WT = 0%; HCN4FEA = 57.1%; Fig. 4h). Cellular capacitances and HCN current densities determined in perforated-patch mode were similar in WT and HCN4FEA cells with permanent and episodic firing, respectively, (WT, permanent firing: −12.6 ± 3.0 pA pF^−1^, *n* = 5; WT, episodic firing: −15.4 ± 4.3 pA pF^−1^, *n* = 4; HCN4FEA, permanent firing: −10.0 ± 3.0 pA pF^−1^, *n* = 4; HCN4FEA, episodic firing: −15.0 ± 2.5 pA pF^−1^, *n* = 6; Supplementary Data 7a, 7c). T-type and L-type calcium current density and response to cAMP were also similar in WT and HCN4FEA cells (Supplementary Data 7d).

### Dynamic mode-shifts in the voltage dependence of HCN4

Compared to firing, nonfiring is characterized by hyperpolarized membrane potentials. To test whether this hyperpolarized potential influences voltage-dependent activation of HCN4 channels, we recorded steady-state activation curves from different holding potentials (HP) corresponding to membrane potentials during the firing (−55 mV) and nonfiring mode (−65 and −75 mV). These experiments revealed that the activation curves and *V*_0.5_ values are strongly dependent on the HP (Supplementary Fig. 3a, Supplementary Data 1a). The most negative *V*_0.5_ value (*V*_0.5_: −100.1 ± 1.9 mV) was obtained for WT channels without cAMP using a HP of −55 mV. *V*_0.5_ values are shifted to more positive values for HPs of −65 (*V*_0.5_: −90.7 ± 4.0 mV) or −75 mV (*V*_0.5_: −77.6 ± 3.1 mV). cAMP shifted *V*_0.5_ values to more positive potentials in WT channels. *V*_0.5_ was −82.1 ± 2.8 mV in the presence of cAMP using a HP of −55 mV and shifted to a more positive value of −62.0 ± 2.8 mV at a HP of −65 mV and even to −51.3 ± 1.7 mV at a HP of −75 mV. For HCN4FEA channels, the *V*_0.5_ values in the absence and presence of cAMP at each holding potential were similar and were positioned in between *V*_0.5_ values obtained in the absence or presence of cAMP for WT channels. Taken together, these results indicate that HCN4 channels are characterized by a history-dependent and dynamic voltage dependence. This phenomenon has been described as hysteresis and mode shift of voltage-dependent channel activation^29–33^.

### Nonfiring pacemaker cells in the SAN network

**Fig. 6:**
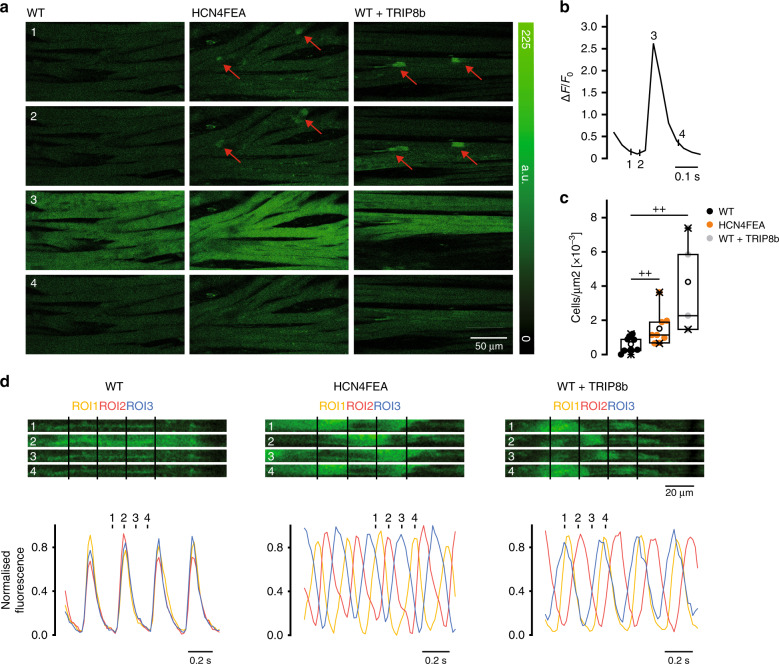
Nonfiring pacemaker cells can be identified in the SAN network. **a** Time-lapse of confocal calcium recordings (Fluo-4 AM; fluorescence in arbitrary units [a.u.]) of intact SAN explants of WT, HCN4FEA, and WT after application of TAT-TRIP8b_nano_ (*n* = 7 WT + 8 HCN4FEA + 4 WT + TRIP8b biologically independent samples). Red arrows indicate subthreshold calcium signals. Images were taken at time points 1–4 during WT calcium transient shown in **b**. **c** Number of cells per µm^2^ displaying subthreshold calcium signals determined from the entire SAN region (*n* = 7 WT + 8 HCN4FEA + 4 WT + TRIP8b biologically independent samples; Mann–Whitney U test; WT vs HCN4FEA: *p* = 0.0330; WT vs WT + TRIP8b: *p* = 0.0040). **d** (upper panel) Time-lapse of confocal calcium recordings of intact SAN explants of WT, HCN4FEA, and WT after application of TAT-TRIP8b_nano_ (*n* = 7 WT + 8 HCN4FEA + 4 WT + TRIP8b biologically independent samples). Images were taken at time points 1–4 indicated in the corresponding calcium transients (lower panel). Ca^2+^ transients were determined from indicated regions of interest (ROI). All experiments were performed using tissue isolated from female animals. Boxplots show the median line, perc 25/75, and min/max value; open symbols represent the mean value. Significance levels: ^+^Mann–Whitney U test. Source data are provided as a Source Data file.

At this point, the question arises of how the nonfiring observed in single pacemaker cells becomes apparent in the SAN network (Fig. 6). To address this question, confocal Ca^2+^ imaging experiments of in vitro preparations containing the SAN network and surrounding tissue were performed in which global Ca^2+^ signals were used as readout for firing and nonfiring states in single pacemaker cells. The vast majority of individual WT pacemaker cells displayed rhythmic and global Ca^2+^ transients (Fig. 6a, d left panels, and Supplementary Movie 1) without exhibiting a nonfiring state. However, in a fraction of HCN4FEA cells, distinct subthreshold Ca^2+^ signals were observed (Supplementary Movie 2). Some of these cells displayed spontaneous and highly localised Ca^2+^ events during diastole and global Ca^2+^ transients during systole (Supplementary Movie 4). Another cell population displayed Ca^2+^ waves propagating from one end of the cell to the other (Fig. 6d middle panel; Supplementary Movie 5) or central Ca^2+^ waves spreading bidirectionally along the long axis (Supplementary Movie 6). These Ca^2+^ signals were self-limiting, did not trigger global Ca^2+^ transients, and were temporally and spatially unrelated to global Ca^2+^ transients of neighbouring cells; this indicates that these cells are indeed nonfiring. Sometimes, Ca^2+^ waves were not restricted to a single cell in the network but were also transmitted to neighbouring cells, resulting in small cell clusters with irregular Ca^2+^ activity (Supplementary Movie 2). This indicates an interaction between these cells and a functional impact of single, nonfiring cells on the SAN network. In WT SANs, localised Ca^2+^ activity and Ca^2+^ waves were rarely observed; however, they could reliably be induced after application of TAT-TRIP8b_nano_ (Fig. 6a, d right panels; Supplementary Movie 3). This confirms that the Ca^2+^ signals are specifically caused by acutely switching off HCN4 CDR. Subthreshold Ca^2+^ activity was never observed during the nonfiring state in isolated single pacemaker cells and only occurred in the SAN network.

### In vitro HR response to vagal nerve stimulation

**Fig. 7:**
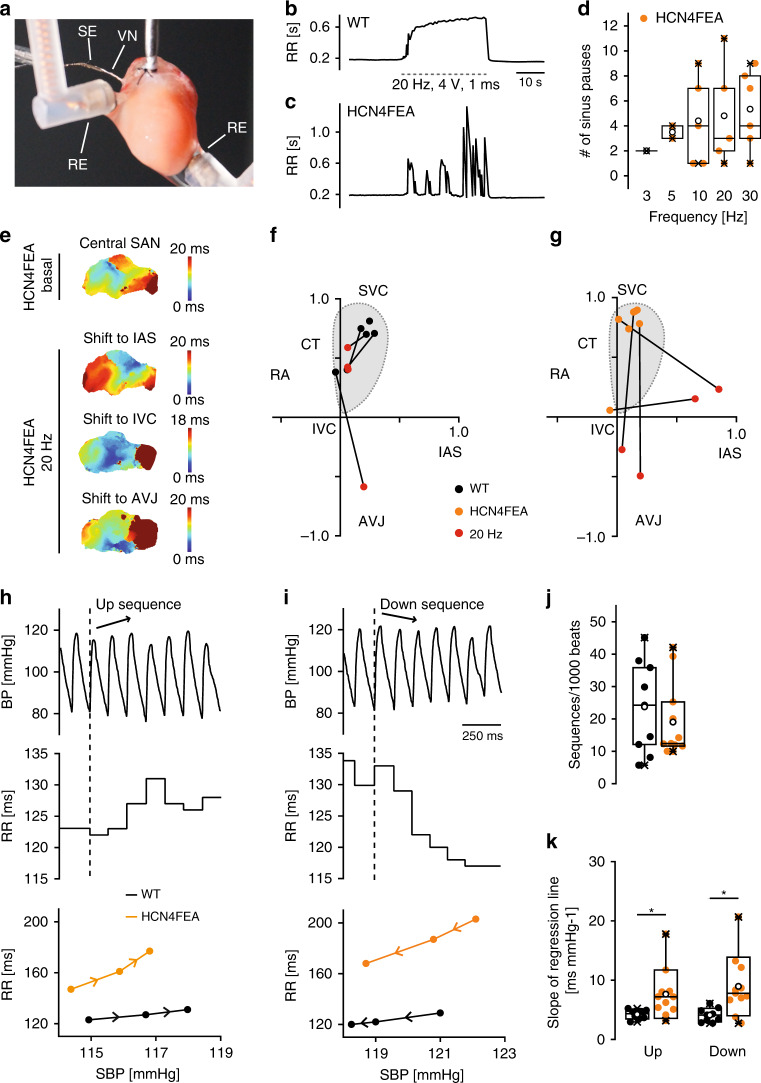
Vagal nerve stimulation in vitro and spontaneous vagal nerve activity in vivo induce overshooting HR responses. **a** Experimental setup consisting of a Langendorff-perfused heart preparation. VN vagal nerve, SE stimulating electrode, RE ECG recording electrodes. **b, c** RR tachograms of **b** WT and **c** HCN4FEA ECGs before, during (dotted lines), and after vagal nerve stimulation (VNS). **d** Mean number of sinus pauses in HCN4FEA hearts at different stimulation frequencies during VNS (3 Hz *n* = 1, 5 Hz *n* = 2, 10 Hz *n* = 5, 20 Hz *n* = 5, 30 Hz *n* = 6 biologically independent samples). Sinus pauses were not observed in WT hearts. **e** Activation map of a HCN4FEA biatrial preparation (top) before and (bottom) during VNS. **f, g** Position of the leading pacemaker in WT and HCN4FEA preparations before and during VNS. IAS: intra-atrial septum; IVC: inferior vena cava. **h, i** Combined telemetric blood pressure (BP) and ECG recordings. **h** (top) Arterial BP trace of a WT mouse during a short sequence of three consecutive beats with increasing systolic BP (up sequence; arrow). **h** (middle) Corresponding RR intervals. **h** (bottom) Plot of systolic BP (SBP) and corresponding RR intervals for a WT and HCN4FEA mouse (see methods for details). **i** BP trace during a short sequence of three consecutive beats with decreasing systolic BP (down sequence). **j** Total amount of up and down sequences (*n* = 9 WT + 11 HCN4FEA animals). **k** Mean slope of the RR/SBP relationship (*n* = 9 WT + 11 HCN4FEA animals; two-sided *t*-test; slope up: *p* = 0.0238; slope down: *p* = 0.0103). In vivo experiments were performed using male animals. In vitro experiments were performed using tissue isolated from female animals. Boxplots show the median line, perc 25/75, and min/max value; open symbols represent the mean value. Significance levels: *student´s paired *t*-test. Source data are provided as a Source Data file.

Loss of HCN4 CDR induced nonfiring of single SAN cells, led to moderate firing fluctuations in in vitro SAN preparations and isolated whole hearts and very pronounced HR fluctuations in vivo (Supplementary Data 4c, 6a, 8a). HR fluctuations in vivo mainly arose from ANS activity (Fig. 3i). To test whether firing rate fluctuations can be induced in vitro by stimulation of the ANS, we utilised in vitro preparations containing the vagal nerve (Fig. 7). The advantage of this experiment is that electrical stimulation of the vagal nerve induces physiological, pulsatile, and transient release of acetylcholine from nerve terminals. Vagal nerve stimulation evoked a pronounced and stable decrease in the firing rate of WT whole-heart preparations (Fig. 7b, Supplementary Data 8b), which persisted throughout stimulation. In contrast, it induced short and inappropriately enhanced bradycardic periods characterized by very slow firing or sinus pauses in HCN4FEA hearts that were frequently interrupted by short periods of recovery to faster HR (Fig. 7c, d). Optical imaging of biatrial preparations (Supplementary Fig. 2a–d) demonstrated that the leading pacemaker region position, which is defined as the region of earliest excitation, was similar, remained stable, and was localised within the anatomical territory of the SAN in WT and HCN4FEA preparations. Application of carbachol shifted the leading pacemaker region towards the interatrial septum, inferior caval vein, or AV junction, but these shifts were similar in WT and HCN4FEA SANs (Supplementary Fig. 2e–f and Supplementary Data 6b). In stark contrast, vagal nerve stimulation doubled shifts of the leading pacemaker in HCN4FEA preparations (Fig. 7e–g and Supplementary Data 6c). Altogether, the findings suggest that CDR of HCN4 channels dampens and antagonises the effect of the vagal nerve.

### In vivo HR response to dynamic vagal nerve activity

Dynamic interaction between vagal activity and the heart in vivo was investigated using combined telemetric blood pressure and ECG recordings (Fig. 7h–k). Therefore, 3-h recordings were screened for up and down sequences^34,35^. Up sequences were defined as a sequential increase in blood pressure over three heart beats (arrow in Fig. 7h) that lead to a reflectory increase in vagal activity, which subsequently slows down HR (increase in RR interval; Fig. 7h). Conversely, blood pressure sequentially drops over three heart beats in down sequences, inducing a subsequent increase in HR (decrease in RR interval; Fig. 7i). Importantly, HR responses mainly reflect rapid changes in vagal activity, while sympathetic activity can be assumed constant. For WT and HCN4FEA mice, a similar amount of total sequences was identified indicating similar vagal tone (Fig. 7j, Supplementary Data 9). Inspection of the graphical relationship between HR and systolic blood pressure revealed a higher amplitude and steeper slope for up and down sequences in HCN4FEA mice compared with WT (Fig. 7k). Overall, this indicates that HCN4 CDR dampens and antagonises vagal effects on the heart, which is functionally relevant for the baroreceptor reflex^34,36,37^.

### In vivo HR response to sympathetic nervous system activation

**Fig. 8:**
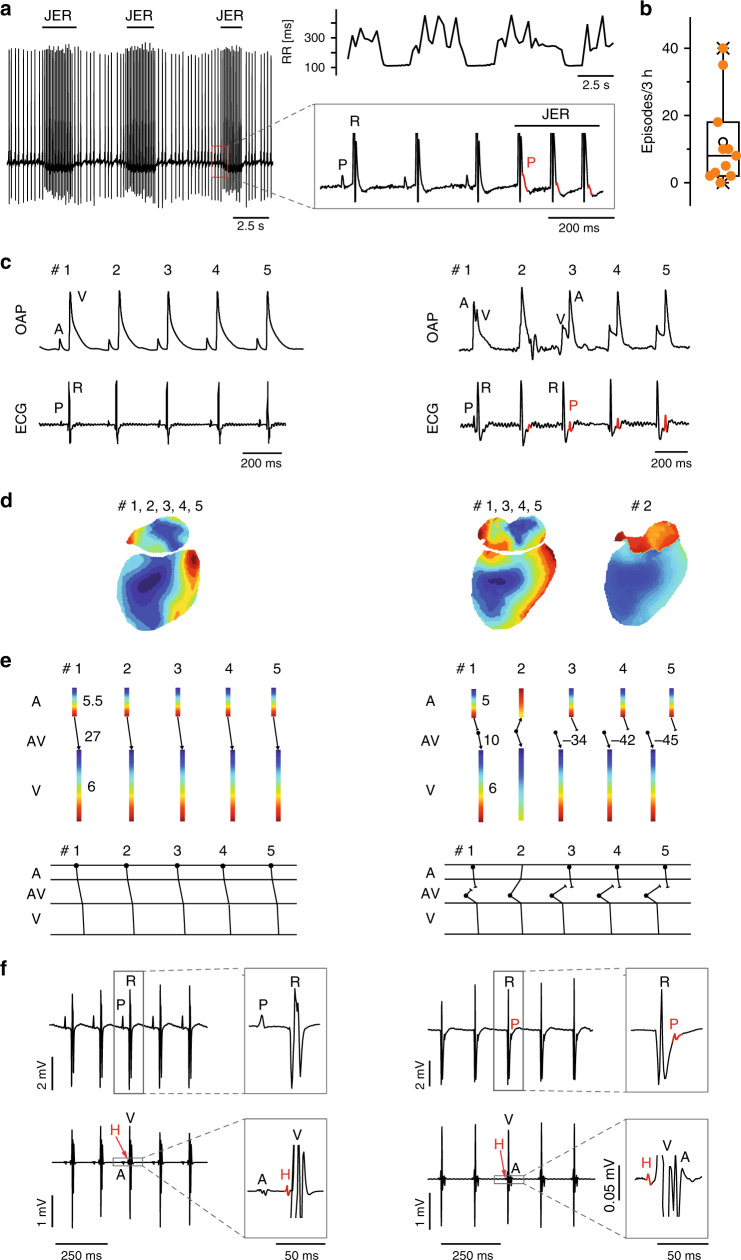
Junctional escape rhythm due to chronotropic incompetence of the HCN4FEA SAN. **a** Telemetric ECG traces from a male HCN4FEA mouse with alternating episodes of sinus rhythm and junctional escape rhythm (JER) rhythm (left) and corresponding RR tachogram (right, top). Inset: During escape rhythm the P wave (red) occurs after the QRS complex. **b** Number of JER episodes during 3 h in low activity phase (*n* = 11 HCN4FEA animals). **c** Optical action potentials (OAP) and surface ECGs recorded from a WT (left) and HCN4FEA (right) Langendorff-perfused heart explanted from female mice. The HCN4FEA heart displays JER. The ventricular signal precedes the atrial signal in beat #3–5. **d** Activation maps determined from the OAP measurements shown in **c**. Right, activation maps for beats #1, and 3–5 were similar. Activation map for beat #2: anterograde activation of ventricles and retrograde activation of atria from the junction. **e** (upper panel) Conduction times determined from **d**. Numbers indicate atrial (top), atrioventricular (middle), and ventricular (bottom) conduction times in ms. Right, Beat 1: ventricles were activated 10 ms after atrial activation. Beat 3–5: ventricles were activated by a subsidiary pacemaker close to the AV junction before atria activation by the SAN. Beat 2: atrial and ventricular activation occurred simultaneously. **e** (lower panel) Schematic ladder diagrams were determined from the measurements shown in **d, e**. **f** Right heart catheterisation of a Langendorff-heart preparation via the superior vena cava from male WT (left) and HCN4FEA (right) animals. Surface (upper panel) and intracardiac ECGs (lower panel). Right, HCN4FEA Langendorff-perfused heart during JER induced by isoproterenol application. Inset: magnification of ECG traces. A His deflection (H, red) precedes the ventricular and atrial signal indicating that the subsidiary pacemaker is localised proximal to the bundle of His. Boxplots show the median line, perc 25/75, and min/max value; open symbols represent the mean value. Source data are provided as a Source Data file.

**Fig. 9:**
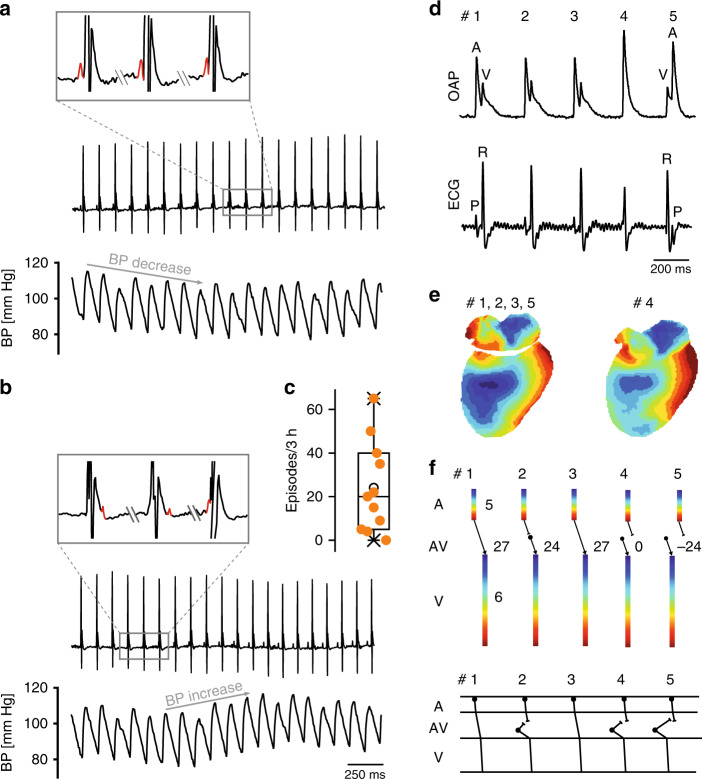
Isorhythmic AV dissociation (IAVD) due to chronotropic incompetence of the HCN4FEA SAN. **a, b** Telemetric ECG trace (top) and corresponding arterial blood pressure recordings **(bottom)** from a male HCN4FEA mouse during IAVD. Characteristic flirtatious P waves indicate the presence of two independent pacemakers (top and inset). Concomitantly with the successive decrease in PR intervals, blood pressure dropped (bottom). **b** (top and inset) The P wave is hidden within the QRS complex, located behind or before the QRS complex. Blood pressure starts to normalise when PR normalises (bottom). **c** Number of IAVD episodes during 3 h in low activity phase (*n* = 11 HCN4FEA animals). **d** OAPs and surface ECG recorded from a female HCN4FEA whole-heart preparation during IAVD. **e** (left) Activation maps of beat #1 determined from the recordings presented in **d** showing IAVD ex vivo. Activation maps for beats 1, 2, 3, and 5 were similar. Right: activation map of beat #4. **f** Ladder diagrams for beats #1–5. Beat #1 and 3: normal activation arising from SAN with regular and constant PR; beat #2, 4, and 5: IAVD with shortened or negative PR indicating that the atria are activated by SAN and ventricles by a subsidiary pacemaker close to the AV junction. Schematic ladder diagrams were determined from the measurements shown in **d, e**. Boxplots show the median line, perc 25/75, and min/max value; open symbols represent the mean value. Source data are provided as a Source Data file.

In HCN4FEA mice, HR acceleration during spontaneous activity of the sympathetic system induced alternating episodes of junctional escape rhythm and sinus rhythm (Fig. 8a, b). Junctional escape rhythm is defined as a specific arrhythmia in which a second, independent, subsidiary pacemaker is present, which is faster than the SAN and thereby suppresses SAN activity. Optical imaging and intracardiac ECG recordings of Langendorff-perfused hearts revealed that the position of the secondary pacemaker was close to the AV junction. This subsidiary pacemaker paces the ventricle and retrogradely activates the atria (Fig. 8c–f, right). In-line with this activation pattern a His bundle potential preceded ventricular and atrial activity in intracardiac ECG recordings of Langendorff-perfused hearts (Fig. 8f, right). Together, these results suggest that the subsidiary pacemaker is localised proximal to the bundle of His and distal to the SAN. When the firing rate of the subsidiary pacemaker approached that of the SAN, isorhythmic AV dissociation occurred in HCN4FEA mice (Fig. 9), which in turn caused oscillations of blood pressure and cardiac output because atrial and ventricular contraction are not properly timed. The presence of isorhythmic AV dissociation and junctional escape rhythm indicates that the coordinated response of the SAN and the AVN (or another subsidiary pacemaker) to changes in ANS activity during HR regulation is impaired in HCN4FEA mice. Furthermore, their presence indicates that the SAN shows chronotropic incompetence (i.e. the SAN cannot accelerate firing rate to maximum rate observed in WT), whereas the chronotropic competence of the subsidiary pacemaker is preserved (i.e. it can accelerate firing rate).

## Discussion

Here we utilized a novel mouse model to investigate the physiological role of HCN4 CDR in the heart. We found that CDR of HCN4 is not required for principal HR regulation by the ANS indicating that the chronotropic response is carried mainly by other ion channels and transporters. In-line with these conclusions are recent studies which also provide evidence that HCN4 channels are not required for the chronotropic response^11,12^. Importantly, our study revealed a complex cardiac phenotype in the absence of CDR characterized by sinus dysrhythmia, severe sinus bradycardia, sinus pauses and chronotropic incompetence, leading to escape arrhythmias, which together reduced cardiac output. Furthermore, the absence of CDR leads to inappropriately enhanced HR responses of the SAN to vagal nerve activity during the baroreceptor reflex. Finally, we discovered a thus far uncharacterised nonfiring mode in single SAN pacemaker cells, which mechanistically can explain the observed phenotype on the single cell level.

**Fig. 10:**
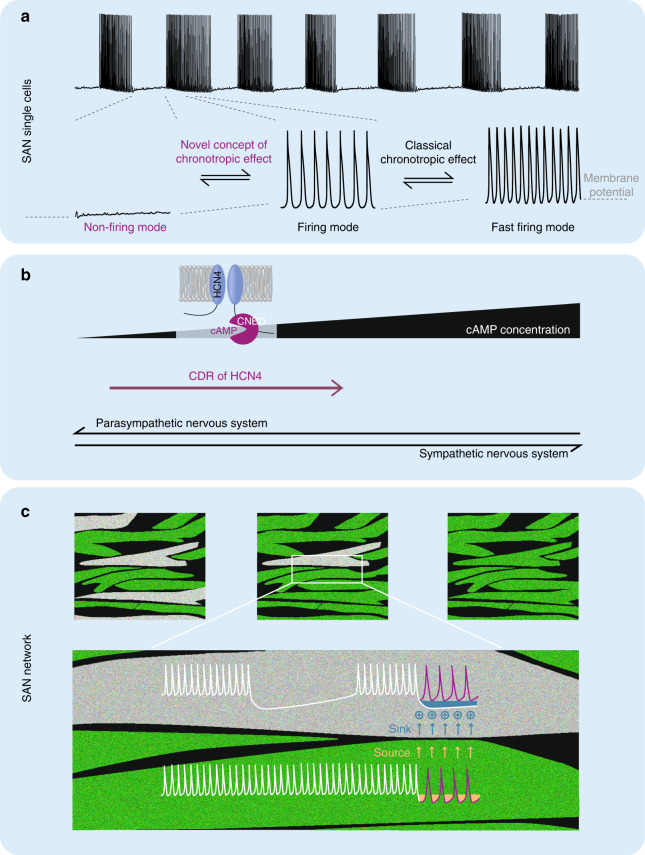
CDR of HCN4 regulates the firing mode of SAN pacemaker cells. **a** (top) Alternating firing and nonfiring episodes in SAN pacemaker cells. During firing cells hyperpolarise and then depolarise during nonfiring. **a** (bottom) Expanded view of nonfiring, slowly firing, and fast firing modes from marked sequences. **b** Illustration of the HCN4 channel. Black ramp indicates cAMP concentration close to the cyclic nucleotide-binding domain (CNBD) which increases from left to right during sympathetic activity. Increased binding of cAMP to the CNBD of HCN4 channels successively switches the activity mode of pacemaker cells from nonfiring (left) to slow firing (middle). Increasing activity of the VN reduces cAMP levels and favours nonfiring. CDR of HCN4 antagonises the response to VN activity and acts synergistically with sympathetic nerve activity. **c** Tonic entrainment process in the SAN network. Pacemaker cells are connected via gap junctions. Nonfiring cells (gray) are more hyperpolarised and draw tonic currents from neighbouring firing cells, which slightly hyperpolarises neighbouring firing cells and depolarises nonfiring cells. Nonfiring cells either remain nonfiring or begin to fire slowly. We suggest that all nonfiring cells form a functional inhibitory cell pool that can be modulated by the ANS via CDR of HCN4. For further details see Discussion.

The nonfiring mode is not only present in HCN4FEA cells but also spontaneously adopted in single WT SAN pacemaker cells (Fig. 10a) and lasts up to a minute. Importantly, this nonfiring mode is by far more pronounced when CDR of HCN4 is lacking. In WT cells, nonfiring can be increased by lowering intracellular cAMP levels by carbachol application or by acute blocking of cAMP binding to HCN4 channels by TAT-TRIP8b_nano._ Reversely, increasing intracellular cAMP by application of isoproterenol markedly reduced nonfiring by shifting nonfiring cells into the firing mode. In-line with the observed induction of nonfiring by carbachol, recent studies revealed that nonfiring can be induced by application of cholinergic drugs in wild-type SAN cells^17,22^ and knockout models in which cholinergic signalling is exaggerated, e.g. the RGS4^18^ or RGS6^19^ knockout mice. In summary, CDR of HCN4 is critically important for maintaining firing and for restoring firing in pacemaker cells that adopt the nonfiring mode. In contrast, lack of CDR leads to reduced stability of firing and thus pacemaker cells spontaneously switch to and remain in the nonfiring mode (Fig. 10a–b).

Our results suggest the following model, which explains the rhythmic changes of firing and nonfiring (Supplementary Fig. 3; note that for the physiological model all values are corrected for liquid junction potential). One important prerequisite for this model is that kinetic parameters of HCN4 (and also other HCN channel subtypes^38^) are much slower than the duration of the pacemaker potential. Therefore, HCN4 currents can be assumed as being almost constant during the pacemaker potential with only minor and low amplitude oscillations around a constant mean. Thus, the effective voltage, which determines overall HCN4 currents during firing is the average voltage of a pacemaker potential (pink line in Supplementary Fig. 3c). A typical cycle of firing and nonfiring is shown in Supplementary Fig. 3c. Firing starts at a maximum diastolic potential of −67 mV and slowly declines to −75 mV. At the same time, the average membrane potential declines from −51 mV (beginning of firing; pink curve) to −59 mV (end of firing; pink curve). When firing terminates, membrane potential abruptly drops to −75 mV (pink curve). During nonfiring the membrane potential slowly rises to −67 mV until firing is reinitiated, which leads to an abrupt increase in average potential to −51 mV. We propose that during firing and nonfiring pronounced shifts in the activation curves of HCN4 channels occur (Supplementary Fig. 3b), which are driven by the large voltage steps at the beginning and the end of firing (step-like jumps in the pink curve). We suggest that by the end of the firing mode (−59 mV) the activation curve of HCN4 is positioned to more hyperpolarised potentials (black curve, Supplementary Fig. 3b). After the abrupt jump to −75 mV at the beginning of nonfiring the activation curve shifts towards more depolarised potentials (red curve). Once the threshold for firing is reached pacemaker cells switch to firing mode and voltage abruptly jumps to −51 mV shifting voltage-dependent activation to more hyperpolarized potentials (black curve) and the cycle repeats. Voltage-dependent activation of HCN4 is therefore a dynamic and history-dependent process^29–33^. Given the voltage-dependent hysteresis of HCN4 channels^31,33^, the shifts in activation curves lag behind the rather fast voltage jumps due to the slow activation and deactivation time constants of the HCN4 channels. The slow hysteresis of HCN4 maintains firing until the shift of the activation curve to the left is completed. Analog reasoning applies to nonfiring. Together, hysteresis behaviour and slow kinetics of HCN4 channels match slow changes of firing and nonfiring. During firing, HCN4 currents slowly decline and set the timepoint at which firing is terminated. By contrast, HCN4 currents do not influence SDD or firing rate during firing mode.

In WT channels, cAMP/isoproterenol shifts activation curves to more depolarised potentials. It thereby decreases the frequency and length of nonfiring by changing the timepoint at which firing is initiated and terminated. In HCN4FEA cells nonfiring is observed more frequently than in WT with high cAMP (isoproterenol) and with basal cAMP. Also, the duration of nonfiring is longest in HCN4FEA cells, indicating that in the absence of CDR activation curves cannot be sufficiently shifted to depolarised potentials and hence currents cannot be increased to a sufficiently high level to reduce or suppress nonfiring. Finally, our data indicate that basal cAMP levels actually might shift activation curves of WT channels to slightly more depolarised *V*_0.5_ values as compared to the HCN4FEA mutant.

Our data show that shifts in *V*_0.5_ parallel respective changes in time constants. Importantly, activation time constants of HCN4FEA channels expressed in HEK293 cells are in between the respective values obtained for WT channels in absence of cAMP and presence of saturating cAMP concentrations (order of activation time constants: WT w/o cAMP > HCN4FEA w/o cAMP = HCN4FEA cAMP > WT cAMP). Deactivation time constants of HCN4FEA are similar to WT under conditions without cAMP (order of deactivation time constants: WT w/o cAMP = HCN4FEA w/o cAMP = HCN4FEA cAMP < WT cAMP). Assuming simple Hodgkin-Huxley formalism for HCN4 channels, one would expect that the peaks of the kinetic-voltage-relationship follow the same order as respective *V*_0.5_ values. Shifts of *V*_0.5_ and peaks of the kinetic-voltage-relationship to more positive potentials together with faster activation and unchanged deactivation in the mutant increase availability of HCN4FEA channels as compared to WT channels without cAMP. However, in the presence of cAMP, the availability of HCN4FEA and the constitutive current is smaller as compared to WT HCN4 channels, because *V*_0.5_ and the peak of the kinetic-voltage-relationship are less positive, the activation time constant is slower and the deactivation constant faster. Lower availability of the mutant versus the wild-type could lead to nonfiring more easily. While this explains how intermittent firing might arise more frequently in HCN4FEA compared to WT pacemaker cells, future experiments and modelling studies need to be performed to complement and confirm this.

In addition to mode-shifts and kinetic-related issues, other factors could contribute to intermittent firing. Possible explanations include slow oscillations of cAMP, which could directly change CDR and thereby the activity of HCN4. In addition, slow intracellular Ca^2+^ rhythms could stimulate Ca^2+^-dependent K^+^ channels, which then induce hyperpolarization and induce nonfiring in SAN pacemaker cells. At the same time, Ca^2+^ could activate Ca^2+^-dependent adenylyl cyclases which in turn increase cAMP levels, activate CDR of HCN4 leading to an delayed increase in HCN4 activity which then effectively opposes the hyperpolarizing effect of K^+^ channels and terminates the nonfiring cycle.

In the SAN network, pacemaker cells are electrically coupled via gap junctions. If a single SAN cell fires slower than the network rhythm, it is briefly depolarized during its slow diastolic depolarisation phase by action potentials fired in surrounding cells. This depolarization is caused by transient inter-cellular currents that pass through gap junctions and elicits an action potential in the slower cell to bring it back to the network rhythm. Vice versa, if a single pacemaker cell is faster than the SAN network rhythm it is slowed by neighbouring cells. These processes are known as mutual, phasic entrainment. By contrast, the interactions between nonfiring cells and firing cells last longer and are therefore assumed to be tonic (Fig. 10). We suggest the term tonic entrainment in order to specifically discuss the interaction between firing and nonfiring cells. The assumption is, that whenever a pacemaker cell in the SAN network hyperpolarizes in order to enter the nonfiring mode this cell would electrotonically draw tonic flows of cations from more depolarised firing cells via gap junctions (Fig. 10c). As a consequence, firing cells would slightly hyperpolarize and fire more slowly and the nonfiring cell would slightly depolarize. Thus, a bradycardic network rhythm emerges, which is slower than the intrinsic firing rate of firing cells. Finally, the cation flow caused by the tonic entrainment process would redistribute Ca^2+^ ions towards nonfiring cells, giving rise to subthreshold Ca^2+^ activity. Our model predicts that in the WT SAN the balance between inhibition (nonfiring cells) and excitation (firing cells) is stabilised by CDR of HCN4. In the absence of CDR, this balance is shifted towards inhibition. If too many cells are in the nonfiring mode, impulse formation and conduction is slowed down, and bradycardia and chronotropic incompetence arise. The presence of slow and fast-conducting SAN cells side by side induces a dispersion in conduction velocity and discontinuous conduction, contributing to fluctuations in firing rate and intrinsic sinus dysrhythmia. The tonic entrainment might be similar to interactions between atrial cells, which have a more hyperpolarized resting membrane potential than pacemaker cells, and SAN pacemaker cells at atrio-sinoatrial contact sites^20,39–42^.

How does the tonic entrainment fit to the classical mechanism of the chronotropic effect? According to the classical view of the chronotropic effect, SAN pacemaker cells are continuously firing and the ANS induces the chronotropic response by modulation of the firing frequency (Fig. 10a; classical chronotropic effect). By considering nonfiring pacemaker cells in the SAN this model can be extended (Fig. 10a; novel concept of chronotropic effect). All cells that are nonfiring at a given moment contribute to an inhibitory pool of cells within the SAN, which is able to slow down firing in neighbouring firing cells by tonic entrainment. Activation of the vagal nerve increases the number of nonfiring cells and thereby also increases the impact of this inhibitory cell pool, whereas CDR of HCN4 can effectively counteract this effect. Increasing sympathetic activity gradually reduces the population of cells in the nonfiring mode (Fig. 10a; nonfiring to firing mode) and subsequently increases activity of firing cells (Fig. 10a; firing to fast firing mode). Thus, the role of CDR of HCN4 during activity of the sympathetic nervous system is to drive nonfiring cells to the firing mode. Furthermore, CDR sets the average HR, and shifts the position of the HR range to higher HR values. Reversely, in the absence of CDR, the average HR and the full HR range is shifted towards lower HR values (Fig. 3d). Furthermore, CDR opposes inappropriately enhanced responses to vagal activity, which tend to shift more firing cells into the nonfiring mode and thereby lead to severe bradycardia and sinus pauses. Finally, by adjusting the balance between firing and nonfiring cells CDR stabilizes the HR by reducing HR fluctuations. Together, the effects described above define novel roles of CDR of HCN4 within the framework of the chronotropic effect.

Our model also explains the shifts in the leading pacemaker region in response to activation of the ANS. The role of CDR is most pronounced in the head region of the SAN, where HCN4 expression is highest. In this region, the leading pacemaker is localised at baseline and during high activity of the sympathetic nervous system. Upon activation of the vagal nerve, cells in the head region of the SAN preferentially switch to nonfiring mode, slowing down firing. This effect is less pronounced in the more peripheral parts of the SAN and at the AV junction where HCN4 expression is lower. As a result, pacemaker cells close to the AV junction fire faster than cells in the head of the SAN and the leading pacemaker region shifts downwards towards the AV junction. In the absence of CDR, the response to vagal nerve activity is more pronounced and larger shifts occur.

In vivo and in vitro, vagal nerve activity leads to inadequately enhanced HR responses in HCN4FEA, indicating that in the absence of CDR of HCN4, the SAN is highly susceptible to perturbations induced by the ANS, giving rise to pronounced HR fluctuations, which clinically manifest as sinus dysrhythmia. Conversely, CDR of HCN4 is required to dampen and counteract HR-lowering vagal effects. These properties fit the role of HCN channels in stabilising and dampening transient changes in membrane potentials and firing rates of the SAN^1,20^. Together, our results indicate that while the tone and dynamic regulation of the ANS is unchanged (e.g. number of up and down sequences are equal and full ranges of spontaneous HRs and blood pressure regulation are preserved in telemetry), inadequately enhanced HR responses arise from an intrinsic change in responsiveness of the SAN caused by lack of HCN4 CDR.

Finally, the concerted response of the SAN and AVN during chronotropic response is disrupted in the absence of CDR. While the SAN is bradycardic and unable to accelerate the firing rate to the maximum values observed in wild-type in the absence of CDR (i.e. the SAN is chronotropically incompetent), the AVN is chronotropically competent, possibly due to lower expression of HCN4 and the presence of other mechanisms that independently accelerate firing of the AVN. In-line with this, HCN4 channel expression and I_f_ is lower and I_Ca_ is higher in AVN as compared to the SAN^11,21,43,44^. Therefore, I_Ca_ could provide an independent mechanism for accelerating the firing rate in the AVN. During sympathetic activation, the SAN firing rate frequently lags behind that of the AVN leading to escape phenomena, such as isorhythmic AV dissociation and junctional escape rhythm. When the AVN firing rate approaches and roughly matches that of the SAN, isorhythmic AV dissociation occurs. When the AVN firing rate exceeds that of the SAN, junctional escape rhythm occurs (Supplementary Fig. 4).

Sinus dysrhythmia and chronotropic incompetence are clinically highly relevant and well-known manifestations of the sick sinus syndrome in humans, which is connected to susceptibility to sudden cardiac arrest and sudden cardiac death. While mouse and human absolute HR values differ, the relative dynamic range of HR regulation and the underlying mechanisms are similar, corroborating the general relevance and validity of our findings for cardiac function and disease in human patients. We demonstrated that cells in the pacemaker region can stop firing for up to a minute, which is in stark contrast to intuitive knowledge that our heart is continuously beating. Furthermore, we showed that CDR regulates the concerted action on nonfiring and firing cells during the chronotropic response. The major overall benefit of regulating nonfiring cells is that heartbeat precision and the dynamic range of HR regulation are markedly elevated. In conclusion, CDR of HCN4 is essential for protecting the heart during destabilising and harmful activation of the ANS, but is not crucial for changing HR. Stable and precise HR control via ANS is of major clinical importance during development and progression of cardiovascular disease, including hypertension, heart failure, arrhythmias, and sudden cardiac death.

## Methods

### Ethics statement

All animal studies were approved by the Regierung von Oberbayern, were in accordance with German laws on animal experimentation, and were performed in compliance with widely accepted ethical standards. Effort was taken to keep the number of animals at a minimum.

### Generation of the HCN4FEA mouse line

Using homologous recombination in ES cells we generated a global knockin mouse model (HCN4FEA) that expresses HCN4 channels with two amino acid substitutions in the cyclic nucleotide-binding domain (R669E, T670A) and one substitution in the C-linker (Y527F, Fig. 1e). The targeted HCN4 WT locus comprises exons 3-8. The C-linker mutation is located in exon 4 and both cyclic nucleotide-binding domain mutations are located in exon 7.

### Animal studies

WT and HCN4FEA mice (*Hcn4*^tm3(Y527F;R669E;T670A)Biel^) were obtained by in-house breeding and maintained on a mixed C57BL/6 N and 129/SvJ background. Breedings of one male and two female animals were kept in conventional Eurostandard Type III cages under SPF conditions in a 12 h dark-light-cycle environment with food and water ad libitum. Ambient temperature was 22 °C and humidity 60%. At P21 offspring were separated and group housed (2–5 animals/cage; Eurostandard Type II Long) under the same environmental conditions. Animals were kept for 2–6 months and littermates of the same sex were randomly assigned to experimental groups as indicated at the respective paragraph in the Method Details section.

### Cell lines

HEK293 (human [*Homo sapiens*] embryonic kidney) cells were obtained from ATCC [https://www.atcc.org/] and cultured in Dulbecco´s Modified Eagle Medium (DMEM, low glucose, GlutaMax^TM^ Supplement, pyruvate) supplemented with 10% fetal bovine serum, 100 I.U. ml^−1^ penicillin, 100 µg ml^−1^ streptomycin, and 1.0 mg ml^−1^ G418. Flp-In™-293 (human [Homo sapiens] embryonic kidney) cells were obtained from Thermo Fisher Scientific and stable cell lines were generated using the Flp-In™ system from Invitrogen (Thermo Fisher Scientific, USA). Flp-In™-293 cells were cultured in DMEM (high glucose, GlutaMax^TM^ Supplement, pyruvate) supplemented with 10% fetal bovine serum, 100 I.U. ml^−1^ penicillin, 100 µg ml^−1^ streptomycin, and 100 µg ml^−1^ hygromycin B. All cell lines were incubated at 37 °C with 10% CO_2_.

### RNA isolation, ChIP processing, and bioinformatics analysis

RNA from SAN of male WT and HCN4FEA mice (*n* = 3 each) was isolated using the RNeasy Micro Kit (Qiagen, Netherlands). Quality of RNA specimen was checked on an Agilent BioAnalyzer 2100 (Agilent Technologies, Germany) and processed for Affymetrix Gene Chips using Affymetrix whole transcript sense target labeling kit (Affymetrix, USA). Fragmented and labeled cDNA was hybridized onto murine MouseGene1.1-ST Gene Chips. Staining of biotinylated cDNA and scanning of arrays were performed according to the manufacturer’s recommendations. Raw CEL-files were imported into Expression Console 1.0. RMA (Affymetrix, USA), which was used for array normalization and signal calculation. Normalized signal values were imported into Partek Genomics Suite 6.5. The probe sets were used to calculate differentially expressed transcripts using Welch’s *t*-test with a *p* value cutoff of 0.05 and a fold-change of 1.5.

### RNA isolation and quantitative RT-PCR

RNA from SAN of 3-month-old WT and HCN4FEA mice (*n* = 3 each) was isolated using the RNeasy Micro Kit (Qiagen, Netherlands). cDNA was synthetized with the RevertAid First Strand cDNA Synthesis Kit (Thermo Fisher Scientific, USA). RT-PCR was performed as described previously^9^ and was repeated three times.

### PyMOL

The figure of the C-terminus structure was prepared using PyMOL. The structure is based on sequence 521–723 of the human HCN4 structure in complex with cAMP^45^, downloaded from the protein data base (accession code: 3U11).

### Conventional electrophysiology in HEK293 cells

HEK293 cells transiently expressing WT HCN4 or mutated HCN4FEA channels were used for recordings. Currents were measured at room temperature (RT) using the whole-cell voltage-clamp technique with an Axon 200B amplifier and Clampex 10.5.2.6 software and analyzed with Clampfit 10.5.2.6 software. The extracellular solution was composed of 135 mM NaCl, 5 mM KCl, 1.8 mM CaCl_2_, 0.5 mM MgCl_2_, and 5 mM HEPES (pH adjusted to 7.4 with NaOH). The intracellular solution contained 130 mM KCl, 10 mM NaCl, 0.5 mM MgCl2, 1 mM EGTA, 5 mM HEPES, 3 mM MgATP, and 0.5 mM NaGTP (pH adjusted to 7.4 with KOH). 100 µM cAMP was added to the solution and the pH was readjusted. Steady-state activation curves were determined by hyperpolarizing voltage steps from −140 to −30 mV in 10 mV increments from a holding potential of −40 mV for 4.5 s (pulse interval of 20 s) followed by a step to −140 mV. To reflect more physiological conditions, steady-state activation curves were also measured at 30 °C from more hyperpolarised holding potentials (−55 mV, −65 mV, −75 mV) and voltage steps from −140 to 0 mV were applied in 10 mV increments for 5.0 s (pulse interval of 30 s) followed by a step to −140 mV for 0.5 s. Currents measured immediately after the final step to −140 mV were normalized to the maximum current (*I*_max_) and plotted as a function of the preceding membrane potential. The data points were fitted with the Boltzmann function: (*I* − *I*_min_)/(*I*_max_ − *I*_min_) = (1 − exp((*V*_m_ − *V*_0.5_)/*k*)), where *I*_min_ is an offset caused by a nonzero holding current, *V*_m_ is the test potential, *V*_0.5_ is the membrane potential for half-maximal activation, and *k* is the slope factor.

To determine activation and deactivation kinetics, WT HCN4, HCN4F, and HCN4FEA channels stably expressed in Flip-In™-293 cells were measured at RT using the whole-cell voltage-clamp technique. For determination of activation kinetics the same solutions as described above were used, whereas for determination of deactivation kinetics potassium concentration in the extracellular solution was increased to 30 mM by equimolar replacement of NaCl with KCl. For cAMP supplemented measurements 100 µM cAMP was added to the intracellular solution and the pH was readjusted. Activation and deactivation kinetics were determined after hyperpolarizing the cell to −140 mV for 3.75 s from a holding potential of −40 mV, with subsequent voltage steps back to −30 mV to −60 mV applied for 9 s. For activation kinetics current traces at potentials ranging from −140 mV to −110 mV and for deactivation kinetics current traces at −30 mV to −60 mV following maximal hyperpolarization were fit with a single exponential function (*I* = *I*_ss_ + *A* exp[−*t*/τ]) after an initial delay. *I*_ss_ represents the steady-state current, and τ represents the time constant. Linear leak current was subtracted.

### Gelatine-inflated hearts

Gelatine-inflated hearts were prepared as described previously^1^. The mouse was anesthetized with 5% isoflurane (CP-Pharma, Germany) inhalation and sacrificed by cervical dislocation. The chest was opened and several incisions were made into the liver. The heart was perfused via the left ventricle with PBS until it was free from blood, followed by perfusion with warm 2% aqueous gelatine solution. Immediately, cold PBS was poured onto the gelatine-filled heart and the whole body was stored at 4 °C for 1 h to let the gelatine solidify. The heart was then carefully excised and transferred into a dish containing cold PBS to remove excess gelatine and tissue. Images were taken using a stereomicroscope (Stemi 508, Carl Zeiss AG, Germany) equipped with a color camera (AxioCam 512 color, Carl Zeiss AG, Germany) and ZenCore 5.3 software.

### Echocardiography

Echocardiographic images were obtained using an ultrasound imaging system for rodents (Vevo 2100, FUJIFILM VisualSonics, Canada), utilizing the systems 22–55 MHz transducer (MS550D). Prior to the measurements, male mice were sedated by inhalation of isoflurane (Abbott, Germany). After achieving sedation, the animals were placed on the systems mouse handling table to monitor body temperature, heart rate, and respiratory rate. Long axis, M-Mode, and PW-Doppler images were taken and analyzed using the system software. During experiments and analysis, the investigators were blinded to genotype and experimental group. Animals were identified by earmarks.

### HE staining

Hearts from 3-month-old male WT and HCN4FEA mice were removed, fixed in 4% paraformaldehyde for 2 h and incubated in sucrose at 4 °C overnight. Hearts were then embedded in optimum cutting temperature compound (Tissue Tek, Sakura Finetek, Germany) and cut into 12-µm thick sections using a cryostat. Sections were stained with hematoxylin and eosin according to standard protocols.

### SAN cross section immunohistochemistry and fibrosis analysis

Three-month-old male animals were sacrificed and hearts were perfused with PBS as described in the gelatine-inflated hearts section. After PBS perfusion the hearts were excised and the SAN dissected. The tissue was fixed with 4% PFA in PBS for 25 min, washed with PBS and afterwards incubated for 2 h in 25% sucrose in PBS. The SAN was embedded in optimum cutting temperature compound (Tissue Tek, Sakura Finetek, Germany) and cut into 4-µm thick sections using a cryostat. Sections were permeabilized for 1–1.5 h with 0.5% Triton X-100 in 20% DMSO in PBS. After washing three times with PBS, blocking was achieved by 1 h incubation in 5% normal donkey serum in PBS. Another three washing steps were followed by overnight incubation at 4 °C with the guinea pig anti-HCN4 and rabbit anti-HCN1 antibodies (1:200 in PBS, Alomone labs, Israel). The slides were washed with PBS before incubation with the secondary antibodies (FITC-conjugated donkey anti-guinea pig, 1:100 and Cy3-conjugated donkey anti-rabbit, 1:400, Merck Millipore, Germany) for 4 h at RT under exclusion of light. Sections were washed, mounted with mounting medium (Vectashield, Vector Laboratories, UK) and analyzed using a Leica SP8 confocal microscope with ×10 magnification. In order to quantify protein distribution of HCN1 and HCN4 in the SAN, ImageJ software was used to analyse fluorescent areas. Thresholds for red fluorescence (HCN1) and green fluorescence (HCN4) were set to ranges that exclude unspecific background signal from surrounding SAN and atrial tissue. The same threshold ranges were used for all images. The remaining SAN area was measured and the ratio of HCN1/HCN4 area was calculated for head, body and tail regions. Consecutive cross sections of the same SAN were incubated overnight in Bouin´s solution, washed for 10 min under running tap water and subsequently stained for 20 min at RT with 0.1% Fast green solution. Slides were rinsed in 1 % acetic acid for 1 min followed by washing for 5 min in tap water. In a last staining step the cross sections were incubated for 30 min in 0.01% Sirius red solution. Finally, slides were dehydrated (70% ethanol, 10 s; 100% ethanol, 3 min; 100% xylole, 3 min) before mounting with Entellan (Merck KGaA, Germany). Images were taken with an Olympus BX41 microscope and cellSens 2.3 software using a ×10 objective. For quantification of red and green areas ImageJ software was used. To assess fibrosis levels, the percentage of red areas in the nodal region was calculated.

### Western Blot

For protein isolation, mouse SAN, atrial, and left ventricular tissue from 3-month-old female WT and HCN4FEA mice was snap-frozen in liquid nitrogen. Samples were homogenized on dry ice using a mortar and pestle and suspended in homogenization buffer (2% sodium dodecyl sulfate, 50 mM Tris and proteinase inhibitor cocktail mix). After heating at 95 °C for 15 min followed by centrifugation at 1000 × *g* for 10 min to remove cell debris, the resulting supernatant was used in western blot analysis as previously described^38^. The following antibodies were used: mouse anti-HCN1 (1:1000; Abcam, UK), rat anti-HCN4 (1:500; Santa Cruz Biotechnology, USA), rabbit anti-HCN2 L (1:500^46^), and mouse anti-beta-tubulin (E7, 1:2000; Developmental Studies Hybridoma Bank, USA).

### Isolation and electrophysiology of mouse SAN cells

Eight- to ten-week-old male WT and HCN4FEA mice were sacrificed by cervical dislocation under deep inhalation anaesthesia consisting of 5% isoflurane (CP-Pharma, Germany) in carbogen (5% O_2_, 95% CO_2_), and death was confirmed by subsequent decapitation. Beating hearts were quickly removed and transferred to warm (37 °C) Tyrode’s solution (Tyrode III) containing: 140 mM NaCl, 5.4 mM KCl, 1 mM MgCl_2_, 1.8 mM CaCl_2_, 5 mM HEPES-NaOH (pH 7.4), and 5.5 mM D-glucose. The SAN region was excised and 2–3 incisions were made to increase the surface for optimized enzymatic digestion. The tissue was then transferred into a ´low-Ca^2+^-low-Mg^2+^´ (Tyrode low) solution containing: 140 mM NaCl, 5.4 mM KCl, 0.5 mM MgCl_2_, 0.2 mM CaCl_2_, 1.2 mM KH_2_PO_4_, 50 mM taurine, 5 mM HEPES-NaOH (pH 6.9), and 5.5 mM D-glucose. Digestion was carried out in a dry block heater for 26–28 min (depending on tissue size) at 37 °C and 450 rpm after adding BSA (1 mg ml^−1^, Merck KGaA, Germany), elastase (18.4 U ml^−1^, Merck KGaA, Germany), collagenase B (0.3 U ml^−1^, Roche Diagnostics, Germany), and protease (1.8 U ml^−1^, Merck KGaA, Germany). Subsequently, the SAN was centrifuged at 200 × *g* for 2 min at 4 °C. The supernatant was discarded and following the same centrifugation protocol the tissue was washed twice with Tyrode low and twice with a modified ´Kraftbrühe´ (KB) medium containing: 80 mM L-glutamic acid, 25 mM KCl, 3 mM MgCl_2_, 10 mM KH_2_PO_4_, 20 mM taurine, 10 mM HEPES-KOH (pH = 7.4), 0.5 mM EGTA, and 10 mM D-glucose. After 3–4 h recovery at 4 °C in 350 µl KB medium and further 15 min adaption to RT, SAN cells were mechanically separated by pipetting the tissue 4–8 times. Fifty microliter of cell suspension were placed on a poly-L-lysine coated coverslip in a vapour-saturated incubation chamber and for proper cell attachment sedimentation was allowed for 15 min. The coverslip was then transferred to the recording chamber and by adding Tyrode III cells were stepwise readapted to a physiological extracellular Ca^2+^ concentration. For current-clamp recording, cells were superfused with Tyrode III at 32 °C. For voltage-clamp experiments, cells were superfused with extracellular solution consisting of 140 mM NaCl, 5.4 mM KCl, 1 mM MgCl_2_, 1 mM CaCl_2_, 5 mM HEPES-NaOH (pH 7.4), 5 mM D-glucose, 0.3 mM CdCl_2_, and 2 mM BaCl_2_ at 32 °C. Pipettes were filled with intracellular solution containing: 90 mM potassium aspartate, 10 mM NaCl, 2.0 mM MgCl_2_, 2.0 mM CaCl_2_, 5.0 mM EGTA, 2.0 mM Na_2_-ATP, 0.1 mM Na_2_-GTP, and 5.0 mM creatine phosphate (pH 7.2). For measurements under saturating cAMP concentrations, 100 µM cAMP (Merck KGaA, Germany) was added to the solution and the pH was readjusted. Recording electrodes were fabricated using a WZ DMZ-Universal microelectrode puller (Zeitz-Instruments Vertriebs GmbH, Germany). We applied the whole-cell and perforated-patch variation of the patch-clamp technique as indicated at the respective experiments. For perforated-patch variation, amphotericin B (EDQM, France) was dissolved in DMSO and added to the pipette solution to obtain a final concentration of 200 µg ml^−1^. Data were recorded using a HEKA EPC10 USB double patch-clamp amplifier (HEKA Elektronik, Germany) and Patchmaster v2x90.2 software. Data were analyzed using Fitmaster v2x90.2 software.

HCN steady-state activation curves were determined in whole-cell configuration by hyperpolarizing voltage steps from −150 to −40 mV in 10 mV increments from a holding potential of −40 mV for 4.5 s (pulse interval of 20 s) followed by a step to −150 mV for 500 ms. Linear leak current was subtracted. Activation kinetics were determined as follows. Current traces at −140 mV were fit with a double exponential function (*I* = *I*_ss_ + *A*_1_ exp[−*t*/τ_1_] ^+^ *A*_2_ exp[−*t*/τ_2_]) after an initial delay. *I*_ss_ represents the steady-state current, and τ represents the time constant. The current density was calculated as the steady-state current amplitude at −140 mV divided by the cell capacitance. Linear leak current was subtracted. Time constants representing activation of HCN4 and HCN1 channels were determined from double exponential fits of I_f_ traces activated by a voltage step to −140 mV from a holding potential of −40 mV.

Calcium currents were recorded in sodium free extracellular solution consisting of 120 mM TEA-Cl, 25 mM HEPES, 10 mM 4-AP, 2 mM CaCl_2_, 1 mM MgCl_2_ (TEA-OH pH = 7.4) at 26 °C. Pipettes were filled with intracellular solution containing: 135 mM CsCl, 5 mM EGTA, 5 mM HEPES, 4 mM Mg-ATP, 1 mM MgCl_2_, 0.1 mM Na-GTP (CsOH pH = 7.2). For measurements under saturating cAMP concentrations, 100 µM cAMP (Merck KGaA, Darmstadt, Germany) was added to the solution and the pH was readjusted. Current densities of I_Ca-T+L_ and I_Ca-L_ were determined at a test potential of −50 mV from a holding potential of −90 and −60 mV, respectively. For determination of I_Ca-T_ the peak inward current of I_Ca-L_ was subtracted from I_Ca-T+L_.

Long-term current-clamp recordings of spontaneous firing activity were carried out in perforated-patch variation and gap-free recordings with a duration of at least 5 min were analyzed. Cells were categorized as “episodic firing” when they displayed continuous periods of at least 10 seconds without spontaneous action potential firing, during which the membrane potential remained at physiological values (average MDP ±10 mV). Cells that displayed no such periods were categorized as “permanent firing”. Upon occurrence of an episodic firing pattern the difference in membrane potential between onset and termination of nonfiring episodes was determined (ΔVm). In order to quantify the incidence of the nonfiring mode the percentage of cells displaying the different firing patterns was calculated. The total duration of nonfiring episodes was assessed and expressed as the percentage of time spent in the nonfiring mode. The shortest nonfiring episode included in the statistics was 3.5 s. For the purpose of studying I_f_ current density and the percentage of permanently and episodically firing SAN cells expressing I_f_, spontaneous firing activity was recorded in perforated-patch variation for 15 min during superfusion with Tyrode III at 32 °C. Subsequently, Tyrode III was replaced with extracellular solution containing CdCl_2_ and BaCl_2_, and voltage-clamp experiments were carried out at 32 °C as described before. Dose-response curves for carbachol were determined as follows. Using the perforated-patch variation, the basal firing rate of spontaneously active SAN cells was measured for 1 min during superfusion with Tyrode III solution. Subsequently, the perfusion was switched to Tyrode III containing carbachol (Merck KGaA, Germany) and the concentration was increased stepwise to 10 nM, 100 nM, and 1 µM, followed by washout with Tyrode III. Only cells that regained rhythmic firing upon washout were included in the analysis (data not shown). Each concentration was administered for a duration of 150 s and cumulative dose-response curves were plotted as the firing rate against the respective concentration of carbachol. To study the effect of isoproterenol, the spontaneous firing activity of SAN cells was first recorded in drug-free Tyrode III solution for 150 s. Afterwards, isoproterenol (100 nM) was washed in and action potentials were recorded for 5 further min. The effect of TAT-TRIP8b_nano_ was determined as follows. WT cells were treated with Tyrode III containing 10 µM TAT-TRIP8b_nano_^28^ at RT for 30 min prior to experiments. Following incubation, current-clamp measurements were carried out in perforated-patch variation during superfusion with peptide-free Tyrode III solution within a timeframe of 60 min. During data analysis, the investigators were blinded to genotype. Animals were identified by earmark numbers.

### Telemetric ECG recordings

Litter-matched 5–6-month-old male WT and HCN4FEA mice were anaesthetized (i.p.; 20 mg kg^−1^ xylazine; 100 mg kg^−1^ ketamine) and radiotelemetric ECG transmitters (ETA-F10, Data Sciences International, USA) were implanted into the intraperitoneal cavity. The ECG leads were sutured subcutaneously onto the upper right chest muscle and the upper left abdominal wall muscle, approximately representing ECG lead II. Carprofen (i.p.; 5 mg kg^−1^; twice daily for 5 days) was given for postsurgical analgesia. The animals were allowed to recover for at least 14 days before measurements were performed. Analog telemetric signals were digitized at 1 kHz and recorded by Dataquest A.R.T. data acquisition software (Data Sciences International, USA). Data were sampled over the whole period of the recording in freely moving animals. For pharmacological interventions drugs were injected intraperitoneally after 2 h prerun. Thereafter, ECGs were recorded for 3–24 h. The animals were allowed to recover for at least 48 h between injections. The following drug concentrations were used in [mg kg^−1^]: propranolol: 20; atropine: 1. Data analysts of all in vivo experiments including ECG data were not blinded to genotype because of obvious differences in the ECG traces of WT compared to HCN4FEA animals.

### Combined telemetric ECG and blood pressure recordings

Litter-matched 4-month-old male WT and HCN4FEA mice were anaesthetized (i.p.; 15 mg kg^−1^ xylazine; 100 mg kg^−1^ ketamine; 1 mg kg^−1^ acepromazine). Combined radiotelemetric ECG and blood pressure transmitters (HD-X11, Data Sciences International, USA) were implanted. Briefly, the pressure sensor was introduced into the aortic arch via the left common carotid artery. The ECG leads were sutured subcutaneously onto the upper right chest muscle and the upper left abdominal wall muscle, approximately representing ECG lead II and the transmitter core unit was placed subcutaneously at the left flank. Carprofen (i.p.; 5 mg kg^−1^; twice daily for 5 days) was given for postsurgical analgesia. The animals were allowed to recover for at least 14 days before measurements were performed. Data analysts of all in vivo experiments including ECG data were not blinded to genotype because of obvious differences in the ECG traces of WT compared to HCN4FEA animals.

### Analysis of heart rate and heart rate variability (HRV)

ECG and HRV analysis was performed using ecgAUTO v3.3.5.10 software (EMKA technologies, France). Mean, minimum, and maximum heart rates were determined from continuous recordings over 72 h, 12 h dark cycle, and 12 h light cycle, respectively. To determine differences in HR dynamics, HR histograms were obtained from 72 h intervals by calculating mean heart rate values every 10 s. Heart rate values were binned using 50 equal distributed windows in the range from 150 to 950 bpm. HRV was determined using an analysis based on previous reports^1^. Briefly, for frequency domain analysis, raw ECG strips from low activity phase were manually inspected to confirm stable sinus rhythm. 103 s time series of RR intervals were plotted as tachograms. These tachograms were interpolated by third-degree spline interpolation at 50 ms intervals to create equidistant points suitable for Fast Fourier Transform (FFT). After detrending, FFT was performed using 1024 spectral points and multiplying with three half overlapping Hamming windows and power spectral density plots were determined. For each time segment, the total power (TP) was calculated as the integral sum of total variability after FFT over the frequency range recorded (0–4.0 Hz). In addition, for each time segment, the cutoff frequencies previously determined to be accurate for mice were used to divide signals into three major components, very low frequency (VLF: 0.0–0.4 Hz), low frequency (LF: 0.4–1.5 Hz), and high frequency (HF: 1.5–4.0 Hz). The data obtained for each time segment were averaged. For time domain analysis, 2 h of ECG recordings were recorded during low activity period. Standard deviation (SD) of all normal RR intervals in sinus rhythm (SDNN) and the square root of mean of squared differences between successive normal RR intervals (RMSSD) were calculated from three representative 10 minute intervals. In addition, 20,000 data points from the cleaned RR time series were used for Poincaré Plots. Here, RR intervals (n; *x*-axis) were plotted against the next RR interval (n + 1; *y*-axis).

### Analysis of interaction between vagal activity, HR, and BP

The beat-to-beat series of systolic BP (SBP) and RR interval during low activity phase was screened using ecgAUTO v3.3.5.10 software (EMKA technologies, France) for spontaneous sequences of increases (up sequences) or decreases (down sequences) in SBP associated with parallel changes in RR interval^35^. The length of the sequences included in the analysis was three consecutive beats. The duration of three consecutive beats (~0.3–1.2 s) is short in comparison with the sympathetic drive, which is characterised by very slow fluctuations in the range of 2–10 s^36^. Therefore, sympathetic drive can be assumed constant during the time window of observation. In contrast, changes in vagal drive are in the millisecond range (200–650 ms)^36,37^; therefore, changes in the RR interval during a sequence are almost entirely related to changes in vagal activity^34,36,37^. The delay between systolic BP and RR was 1 beat, the threshold for BP and RR changes was 0.5 mmHg and 2 ms, respectively, and the slope of the regression line from RR/SBP plots had a correlation coefficient >0.85. Sections of ECG traces exhibiting isorhythmic atrioventricular dissociation in HCN4FEA ECG recordings were excluded to avoid misinterpretation of a BP decrease due to arrhythmia.

### Intracardiac electrophysiological study

Litter-matched 2–3-month-old male WT and HCN4FEA mice were anesthetized with isoflurane and standard intervals were measured in 6-limb leads. For intracardiac electrogram recordings we used a digital electrophysiology lab (EP Tracer, CardioTek, Netherlands). The surgical area was locally anaesthetized by subcutaneous injection of xylocaine (1.7 mM kg^−1^). An octapolar 0.54 mm (1.7 French) electrode catheter (CIBer mouse cath, NuMed Inc., USA) was placed via the right jugular vein into the right atrium and ventricle, guided by the morphology of intracardiac electrical signals. The eight electrodes directly contact the endocardial surface of the heart. Bipolar recordings of the atrial and ventricular depolarizations were obtained from adjacent electrodes in the superior right atrium just past the superior vena cava and right ventricular apex, respectively. Standard clinical electrophysiological pacing protocols were used to determine electrophysiological parameters including sinus node recovery time (SNRT), sinoatrial conduction time (SACT), sinoatrial node effective refractory period (SNERP), refractory periods of the atria, the AV node, and the ventricle as well as AV-nodal conduction properties including Wenckebach periodicity (WBP)^20^. Impulses were delivered at twice diastolic threshold (1 mA) using a pulse duration of 1.0 ms. Each mouse underwent an identical pacing and programmed stimulation protocol. The sinus node function was evaluated by indirect measurement of SNRT by pacing for 30 s at various cycle lengths, starting from a pacing cycle length just below the intrinsic sinus cycle length and measuring the duration of the return cycle, which corresponds to the interval between the last stimulation spike and first spontaneous, sinus node triggered atrial activation. After a pause of 60 s the protocol was repeated by progressively reducing the pacing cycle length in 10 ms steps until a pacing cycle length of 80 ms was reached. Rate corrected SNRT (cSNRT) was calculated by subtracting the averaged sinus cycle length (SCL) from SNRT. SACT was indirectly determined by premature atrial stimulation technique, which was carried out as described^20^. The responses are shown in Supplementary Fig. 1b, c. Premature atrial stimuli were introduced via the stimulation electrode during spontaneous sinus rhythm. The entire sinus cycle was scanned by up to 80 extra stimuli. Spontaneous sinus cycle length (A1A1 interval), coupling interval of the premature atrial stimulus (A1A2), the atrial return cycle length (A2A3) and the postreturn cycle length (A3A4) were measured. Supplementary Fig. 1b illustrates the response of the SAN to premature atrial stimulation. Upon progressively decreasing the coupling interval of the premature atrial stimulus (A1A2), the return cycles A2A3 progressively prolongs. The corresponding A2A3 data points fall on the upper diagonal line indicating fully compensatory pauses [A1A2 + A2A3 = 2(A1A1)]. The pause is compensatory because late diastolic atrial depolarizations do not penetrate and reset the SAN before it fires spontaneously. As soon as the coupling interval A1A2 is decreased below a certain point of the spontaneous cycle length, the return cycles A2A3 is no longer fully compensatory. The data points fall below the line of full compensatory pause but remain greater than one expected sinus cycle length (A1A1; horizontal line). In some animals during this phase A2A3s remained constant yielding a plateau. A1A2 plus A2A3 is shorter than twice the A1A1 interval because the premature atrial depolarization penetrates, depolarizes and resets the SAN prior to its next expected spontaneous firing. The postreturn cycle A3A4, which is the first spontaneous cycle after the return cycle, was also plotted (closed circles). By comparing A3A4 with A1A1 intervals it is possible to assess the sinus cycle variability and SAN automaticity. For calculation of SACT we determined the A2A3 interval, which deviates from the diagonal line. The A1A2 interval at this point represents the shortest premature beat interval whose retrograde excitation front does not reach the sinus node, whereas earlier premature beats reset the pacemaker^20^. The return interval A2A3 at this point, minus the spontaneous atrial cycle A1A1, is equivalent to the sum of conduction time from atrium to the sinus node plus from sinus node to atrium. Half of the total sum of conduction time gives the sinoatrial conduction time. This calculation is based on the assumptions that SACT reflects the time for the paced impulse A1 to enter the SAN, which is then reset plus the spontaneous sinoatrial cycle lengths plus the time it takes for the subsequent spontaneous beat to exit the SAN, and that the times into and out of the SAN are approximately equivalent. Atrioventricular nodal refractory period (AVNERP) was evaluated by programmed atrial stimulation. To allow for reasonable stabilization of refractoriness the premature atrial stimulus (S2) was preceded by a train of 8 paced beats (S1). The train of 8 stimuli was applied at a S1S1 cycle length of 100 ms followed by one extrastimulus (S2). The coupling interval S1S2 was stepwise reduced in 2 ms steps to 20 ms. Subsequently, the protocol was repeated after a recovery time of 30 s using S1S1 cycle length of 90 ms and 80 ms. AVNERP was defined as the longest S1S2 pacing interval with loss of AV-nodal conduction. The minimum cycle length required to maintain 1:1 AV conduction, the Wenckebach paced cycle length, and the maximum paced cycle length causing 2:1 AV block were determined for each animal. The ventricular effective refractory period (VERP) was evaluated analogously to AVNERP. The S1S1 intervals were 100, 90, and 80 ms. The coupling interval S1S2 was stepwise reduced in 2 ms steps to 30 ms. Using a similar protocol, the right atrial effective refractory period (AERP) could not reliably be determined due to superposition of atrial and ventricular electrograms at premature coupling intervals below 40 ms. To circumvent this problem the following three-step protocol was used: After applying a train of 8 S1 stimuli, an extrastimulus (S2) was given to induce AV conduction block at an S1S2 coupling interval 5 ms shorter than the determined AVNERP, followed by an increasingly premature S3. AERP was determined as longest S2S3 with absent atrial response. AV conduction curves were determined from data obtained by the AVERP protocol described above by plotting V1V2 or H1H2 intervals versus A1A2. Latency curves were constructed by plotting A2V2 intervals versus A1A2 intervals as in ref. ^20^. Supplementary Fig. 1d shows the responses to premature stimulation in a single experiment. As atrial responses occurred progressively earlier in the cardiac cycle, ventricular intervals also progressively decreased. Up to a coupling interval of approximately 85 ms, the decrease of V1V2 is proportional to that of A1A2 and therefore the points fall on the line, which represents the theoretical curve of no AV conduction delay. For points on this curve V1V2 is equal to A1A2. At shorter A1A2 intervals, V1V2 decrease less than A1A2. The points lie above the line, indicating that AV conduction of the premature impulse A2 is delayed. The point at which the points start to deviate from the line marks the beginning of the relative refractory period of the AV conduction system. At a critical A1A2 interval V1V2 reaches a minimum and then begins to increase, even though the atrial interval shortened further until complete block of conduction of the premature impulse occurred. From this graph, the functional refractory period of the AV conduction system was determined as the shortest V1V2 interval. We also plotted the intervals between His bundle responses (H1H2 intervals) and premature atrial stimulations (A1A2) for the same experiment. The H1H2 intervals correspond exactly to the V1V2 values indicating that the increase in AV conduction time occurring with premature atrial stimulation was confined entirely to the region of the AV node, i.e. between the atrial and His bundle electrogram. Finally, A2V2 latencies were plotted versus A1A2 intervals. As A1A2 intervals decreases, the A2V2 latency increases (Supplementary Fig. 1f). The A2V2 lengthening is slight for relatively long A1A2 intervals and becomes larger as these intervals shorten. The diagonal line indicates A2V2 lengthening equal to the A1A2 shortenings (slope = -1).

### Langendorff hearts

Ten-week-old female WT and HCN4FEA mice were anesthetized using 5% isoflurane in Carbogen (95% O_2_, 5% CO_2_) and sacrificed by cervical dislocation. After decapitation, the heart was carefully excised and placed in oxygenated, warm (37°C) Krebs Henseleit buffer containing 118.5 mM NaCl, 20 mM NaHCO_3_, 4.7 mM KCl, 1.2 mM MgSO_4_, 1.8 mM CaCl_2_, 1.2 mM KH2PO_4_ and 11 mM glucose (pH = 7.3). Immediately, the aorta was cannulated, mounted on a Langendorff apparatus (Basic Langendorff System, Hugo Sachs Elektronik & Harvard Apparatus GmbH, Germany) and retrogradely perfused with Krebs Henseleit buffer at a constant pressure of 80 mmHg. ECGs were recorded (PowerLab 8/35, LabChart 8, Animal Bio Amp, ADInstruments, New Zealand) by placing electrodes on the right atrium and left ventricle. The hearts were equilibrated for ~10 min prior to measurement. Mean basal HR and HRV parameters were determined from 120 s RR tachograms. Right heart catheterization of Langendorff hearts was performed as follows. Three-month-old male WT and HCN4FEA mice were injected intraperitoneally with heparin (100 IU kg^−1^). Ten-minutes post-injection mice were anesthetized and Langendorff hearts were prepared as described above. For intracardiac electrogram recordings we used a digital electrophysiology lab (EP Tracer, CardioTek, Netherlands). An octapolar 0.54 mm (1.7 French) electrode catheter (CIBer mouse cath, NuMed Inc., USA) was placed via the right jugular vein into the right atrium and ventricle. The mounted hearts were equilibrated before starting the measurements. 100 µl of 200 nM isoproterenol in KH buffer was applied via an injection port within a few seconds to induce IAVD. For recording and analysis LabChart8 (ADInstruments, New Zealand) and EP-Tracer_V1.05 software (Schwarzer Cardiotek, Germany) were used. Vagal nerve stimulation in Langendorff-perfused hearts was performed as follows. Three-month-old female WT and HCN4FEA mice were injected intraperitoneally with heparin (100 IU kg^−1^). Ten-minutes post-injection mice were anesthetized with 5% isoflurane and sacrificed by cervical dislocation. A small incision on the right side of the neck gave access to the vagal nerve and a silk suture was tied around the nerve. The hearts combined with the intact vagal nerve were then carefully removed and mounted on the Langendorff apparatus as described before. For nerve stimulation a custom-made Ag/AgCl electrode was used. A pulse width of 1 ms at 4 V was applied for 30 s using the stimulation frequencies 3, 5, 10, 20, and 30 Hz. Between each stimulation a recovery period of 2 min was inserted. For recording and analysis LabChart8 software (ADInstruments, New Zealand) was used. Calculations were done with Origin 2015. A sinus pause was defined as two consecutive beats with an RR interval >two times higher than the mean RR during stimulation. During data analysis, the investigators were blinded to genotype. Animals were identified by earmark numbers.

### Optical imaging

Langendorff-heart preparations of 3-month-old female mice were used for optical imaging and performed as described above, with the following alterations. Briefly, the mice were injected intraperitoneally with heparin (100 IU kg^−1^). Ten-minutes post-injection mice were sacrificed as described above and the explanted hearts were placed in warm (37 °C) Tyrode solution oxygenated with 95% O_2_ 5% CO_2_, and immediately cannulated. The Tyrode solution contained (in mM) 128.2 NaCl, 4.7 KCl, 1.19 NaH_2_PO_4_, 1.05 MgCl_2_, 1.3 CaCl_2_, 20.0 NaHCO_3_, 11.1 glucose; pH = 7.35^47^. The lung, thymus and fat tissue were dissected and removed. The cannula was mounted to a custom-made Langendorff apparatus and the heart was retrogradely perfused and superfused with Tyrode solution passed through a 10 µm filter. Perfusion pressure in the cannula was monitored with the use of a pressure transducer (MLT0699, ADInstruments, New Zealand) and held at a constant pressure of 80 mm Hg as previously described^48^. ECG traces were recorded (PowerLab 8/35, LabChart 8, Animal Bio Amp, ADInstruments, New Zealand) by placing needle electrodes close to the isolated heart in an approximate Einthoven I configuration. To eliminate motion artifacts, 1 ml of blebbistatin (Cayman Chemical Company, USA), dissolved in DMSO (10 mg ml^−1^) and diluted in Tyrode solution to obtain a final concentration of 0.2 mg ml^−1^, was injected slowly through a drug port located close to the perfusion cannula. After equilibrating for 10 min, 1 ml of Di-4-ANEPPS (Merck KGaA, Germany), dissolved in DMSO (1.25 mg ml^−1^) and diluted in Tyrode solution to obtain a final concentration of 37.5 µg ml^−1^, was applied via the same port. After an equilibration time of 15 min Langendorff hearts were mounted. A high-speed complementary metal-oxide-semiconductor (CMOS) camera (MiCAM05 Ultima-L Single Camera System, SciMedia, USA) faced the posterior side of the heart, which allowed for a clear view of the ventricles, the SAN and both atria. The optical apparatus consisted of an excitation light generated by a 150 W halogen light source (MHAB-150W, Mortitex, Japan), a band-pass filter of 531/40 nm and a fluorescence beam splitting system (THT-FLSP Box, SciMedia, USA). The emitted light was filtered by a 600 nm long-pass filter. The acquired fluorescent signals were collected per pixel and digitalized by the manufacturer´s software (MiCAM05 data acquisition software Ver. 2.5.4, Brainvision Inc., Japan). The following settings were used for optical imaging; a sampling rate of 2 kHz, a frame number of 4096, a spatial resolution of approximately 100 µm/pixel (obtained through a condenser lens (50 mm, M5095 + AD, SciMedia) and a 1.6x objective lense (Planapo, SciMedia, USA). Background fluorescence of optical data was automatically removed and the signals were inverted to correspond with action potentials. The manufacturer´s analysis software (BV_Ana Ver. 1604, Brainvision Inc., Japan) as well as a MATLAB-based graphical user interface (ref. ^49^, RHYTHM 2012) were used for data processing. Isochronal activation maps were built to visualize the first activation site, and action potential propagation through the tissue during sinus rhythm, junctional escape rhythm and IAVD in HCN4FEA mouse hearts.

Optical imaging of biatrial SAN preparations followed the same procedure as described above for Langendorff-heart preparations, with the following modification. Briefly, biatrial preparations of 12-week-old female mice were optically mapped under basal conditions and after application of carbachol. After cannulation and perfusion of the heart, the SAN was dissected together with the right and left atrium, the superior caval vein, the inferior caval vein, the AV junction and was pinned to expose the endocardial surface as described previously^50^. The SAN explant was continuously superfused with Tyrode solution (37 °C). Electrical recordings were performed using three needle electrodes to calculate the beating rate and HRV parameters. To perform optical imaging recordings, the same concentration of blebbistatin (Cayman Chemical Company, USA) and Di-4-ANEPPS (Merck KGaA, Germany) were used as described above. Blebbistatin was directly applied to the preparation followed by Di-4-ANEPPS after an interval of 10 min. After 15 min of equilibration the SAN preparation was optically mapped. The location of the leading pacemaker site and SACT were determined^47,51^. After basal measurements carbachol (Merck KGaA, Germany) (1 µM in Tyrode solution) was delivered through the perfusion system for 5–7 min until it reached steady state. The location of the maximum shift of the leading pacemaker was identified and the distance to the leading pacemaker site under basal conditions was calculated and normalized to the size of the preparation.

Biatrial preparations containing the intact right vagal nerve were prepared as the preparations described above, except that the right vagal nerve was preserved. The vagal nerve was stimulated at 4 V, 1 ms pulse width, 20 Hz for 30 s^52^. The location of the leading pacemaker site was identified as described above.

### Confocal calcium imaging of SAN explants

Intact SAN preparations of 12-week-old female WT and HCN4FEA animals were prepared as described above and loaded with the Calcium indicator Fluo-4 AM (Thermo Fisher Scientific, USA) at RT for 45 min. To this end, Fluo-4 AM was dissolved in DMSO (2 mM), diluted 1:1 with Pluronic F 127 (13% in H_2_O) (Merck KGaA, Germany) and added to Tyrode solution to reach an end concentration of 20 µM Fluo-4 AM. Afterwards, the preparations were continuously superfused with Tyrode solution containing blebbistatin (0.2 mg ml^−1^) at 28 °C. Calcium signals from the head, body, and tail region of the SAN were recorded using a Leica SP8 confocal microscope with a 20x water lens objective. Each frame (440 µm²) was recorded for 10 s with an optically pumped semiconductor laser (OPSL) in-line scan configuration (8 Hz scan speed, 28 frames/s). An excitation wavelength of 488 nm was used and emission was collected >500 nm. Cells displaying highly localized and spontaneous Ca^2+^ release during diastole were counted and normalized to the total surface area measured. Image analysis was performed using Leica LasX software. The change in fluorescence intensity (ΔF) was assessed after background subtraction and normalized to baseline fluorescence F_0_. After basal measurements, TAT-TRIP8b_nano_ (15 µM in Tyrode solution) was added to 4 of the WT preparations and after 30 min incubation, tissue samples were scanned as described above.

### Primer list

HCN1 for: CTGCTGCAGGACTTCCCACCA, HCN1 rev: ATGCTGACAGGGGCTTGGGC, HCN2 for: CAGGAACGCGTGAAGTCGGCG, HCN2 rev: TCCAGGGCGCGGTGGTCTCG, HCN3 for: TGGCCATGGACCGGCTTCGG, HCN3 rev: GAGCCAGGCCCCGAACACCAC, HCN4 for: AGGGCCTTCGAGACGGTTGCGC, HCN4 rev: GGCCATCTCACGGTCATGCCG, ALAS for: TCGCCGATGCCCATTCTTATC, ALAS rev: GGCCCCAACTTCCATCATCT.

### Quantification and statistical analysis

Statistical analysis was performed using Origin 2015 (OriginLab Corporation, USA). All data are given as mean ± SEM. N represents the number of animals, preparations or cells as indicated in the Supplementary Data files. For all statistical tests *p* < 0.05 was considered significant (***p < 0.001, **p < 0.005, *p < 0.05, ns = not statistically significant *p* > 0.05). Differences between two groups were analysed by Student’s unpaired two-sample *t*-test. The *p* values were adjusted using the Holm–Bonferroni correction as indicated in the Supplementary Data files. Experiments with two different variables were analysed with two-way ANOVA. When significant, the analysis was followed by Holm–Sidak multiple comparison test. For EPS parameters that were determined at different coupling intervals data were analysed by two-way repeated measures ANOVA based on general linear model (GLM) or mixed-effect model (REML) in case of missing data due to technical problems. For the experiment in which HCN channel activation and deactivation time constants were determined at different test potentials in HEK293 cells, the data were analysed by three-way ANOVA since the measurements include three independent factors. These factors are (1) Genotype (WT and HCN4FEA), (2) Drug (without and with cAMP), and (3) Potential (several test potentials and repeated measurements within the same specimens). Three-way ANOVA tests the interdependencies of the three factors while taking repeated measurements into account. For data, which do not follow normal distribution or for experiments with low n numbers (*n* = 3) we applied the nonparametric Mann–Whitney U test.

### Reporting summary

Further information on research design is available in the Nature Research Reporting Summary linked to this article.

## Supplementary information

Supplementary Information

Description of Additional Supplementary Files

Supplementary Data 1

Supplementary Data 2

Supplementary Data 3

Supplementary Data 4

Supplementary Data 5

Supplementary Data 6

Supplementary Data 7

Supplementary Data 8

Supplementary Data 9

Supplementary Movie 1

Supplementary Movie 2

Supplementary Movie 3

Supplementary Movie 4

Supplementary Movie 5

Supplementary Movie 6

Reporting Summary

## Data Availability

The Microarray dataset generated and analysed during the current study is available in the National Center for Biotechnology Information Gene Expression Omnibus (GEO) repository and is accessible through the GEO Series accession number GSE138086. Any other datasets generated during and/or analysed during the current study are available from the corresponding author upon reasonable request. Source data are provided with this paper.

## Source data

Source Data

## Code availability

A MATLAB-based graphical user interface (RHYTHM 2012) was used for optical imaging data processing. The current version can be downloaded on Github using this link: [https://github.com/rrasheed/rhythm-analysis-software].
