# Supplementary material for: cAMP-dependent regulation of HCN4 controls the tonic entrainment process in sinoatrial node pacemaker cells
Source: Nat Commun. 2020 Nov 3;11:5555. doi: 10.1038/s41467-020-19304-9 (PMC7641277; doi:10.1038/s41467-020-19304-9)
Supplement: Supplementary file 1 — Supplementary Information [file 41467_2020_19304_MOESM1_ESM.pdf]

SUPPLEMENTARY INFORMATION

cAMP-dependent regulation of HCN4 controls the tonic entrainment process in sinoatrial node pacemaker cells.

Table of contents:

Supplementary Figure 1

Supplementary Figure 2

Supplementary Figure 3

Supplementary Figure 4

Supplementary Figure 1

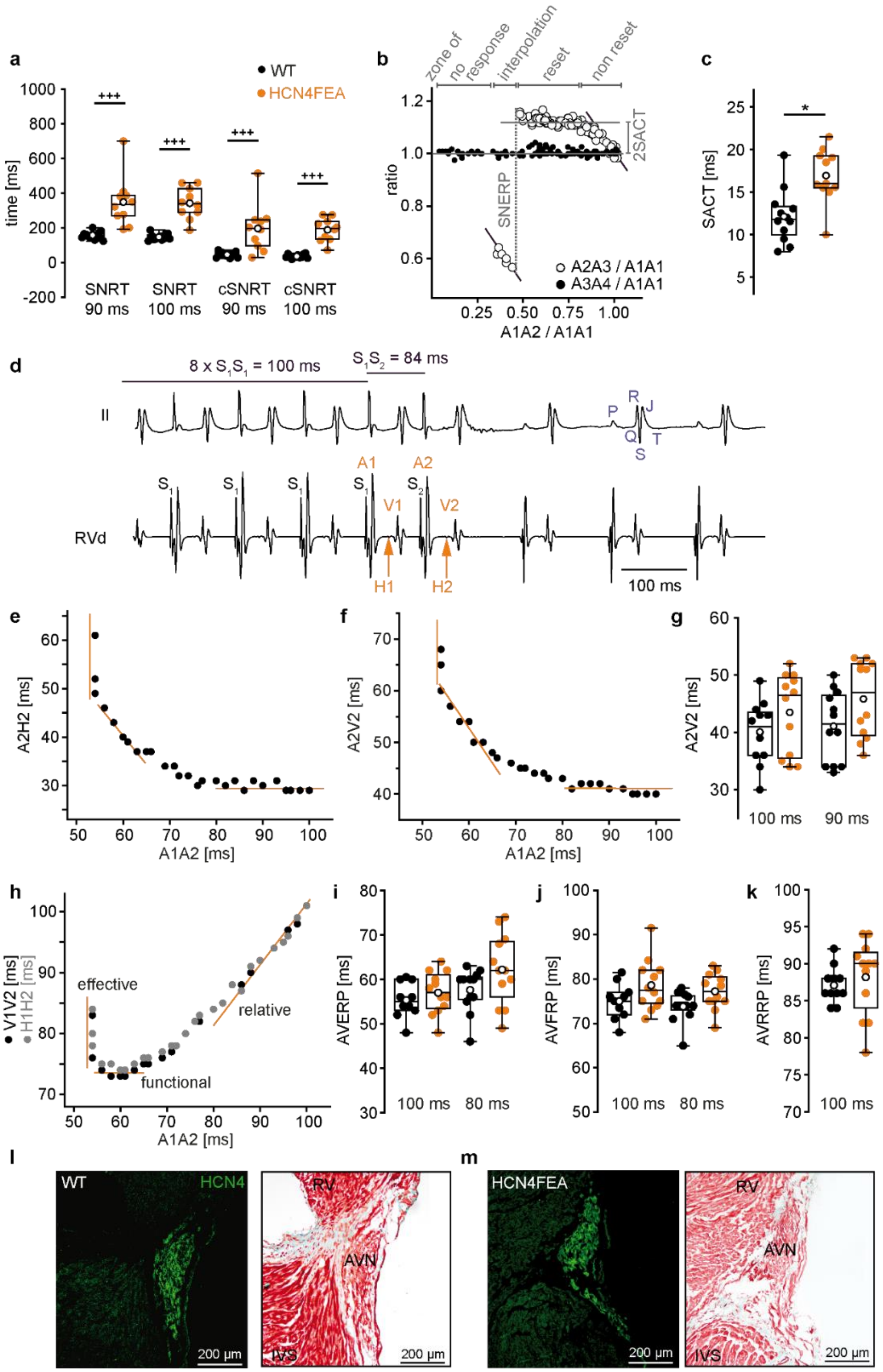

Supplementary Figure 1: Disrupted SAN but preserved AV function in HCN4FEA animals determined via *in vivo* EPS. a, Increased sinus node recovery time (SNRT) in HCN4FEA mice determined from invasive EPS (n = 12 WT + 10 HCN4FEA animals; SNRT90: p=0.00001; SNRT100: p=0.00001; cSNRT90: p=0.00006, cSNRT100: p=0.00005). **b,** Determination of sinoatrial conduction time (SACT) by programmed premature atrial stimulation. The normalised return cycle (A2A3/A1A1) and post return cycles (A3A4/A1A1) are plotted as a function of the normalised coupling interval of the premature atrial stimulus (A1A2/A1A1). Each point represents one test cycle. The diagonal line represents the line of full compensatory pauses [A1A2 + A2A3 = 2A1A1]. **c,** SACT is prolonged in HCN4FEA mice (n = 12 WT + 11 HCN4FEA animals; two-sided t-test, Holm-Bonferroni method: p=0.0075). **d,** AVN function and AV conduction was analysed by premature atrial stimulation. A train of eight stimuli (S1) was applied at S1S1 cycle lengths (100 ms or 80 ms) followed by a premature extra-stimulus (S2) at decreasing S1S2 coupling intervals. Top trace: surface ECG, lead II; bottom trace: intracardial atrial electrogram. S: pacing stimulus artefact; A: atrial signal; H: His bundle signal; V: ventricular signal. **e and f,** AV nodal latency curves were determined by plotting A2H2 or A2V2 delay versus A1A2 intervals. The horizontal line represents the shortest A2V2 interval shown in **(g)** (n = 12 WT + 12 HCN4FEA animals). **h,** AV nodal conduction curves were generated by plotting V1V2 intervals and H1H2 intervals versus the A1A2 coupling interval of the premature atrial stimulus. **i-k,** From AVN conduction curves, AVN effective refractory period (AVERP; 2-way repeated measures ANOVA, mixed effects model REML), functional refractory period (AVFRP; 2-way repeated measures ANOVA, mixed effects model REML), and relative refractory period (AVRRP; two-sided t-test, Holm-Bonferroni method) were determined. Furthermore, AVN function was evaluated using data obtained by telemetric ECG recordings. Analysis of telemetry data revealed that AV conduction was normal in HCN4FEA mice compared with that of WT mice (PQ interval, WT: 38.1 ± 0.8 ms, n = 9; HCN4FEA: 35.2 ± 1.2 ms, n = 11; p = 0.06; Supplementary Data 4). **l, m, (Left)** Cross-sections of the AVN demonstrate similar expression and distribution of HCN4 in WT **(l)** and HCN4FEA **(m). (Right)** Consecutive sections were additionally stained by masson’s trichrome (n = 3 WT + 3 HCN4FEA biologically independent samples). For *in vivo* EPS and cross-sections male mice were used. Boxplots show the median line, perc 25/75, and min/max value; open symbols represent the mean value.

Significance levels: *student´s paired t-test adjusted for multiple comparisons by Holm-Bonferroni correction; + Holm's-Sidak post-hoc test after two-way ANOVA for repeated measures (general linear model). Source data are provided as a Source Data file.

Supplementary Figure 2

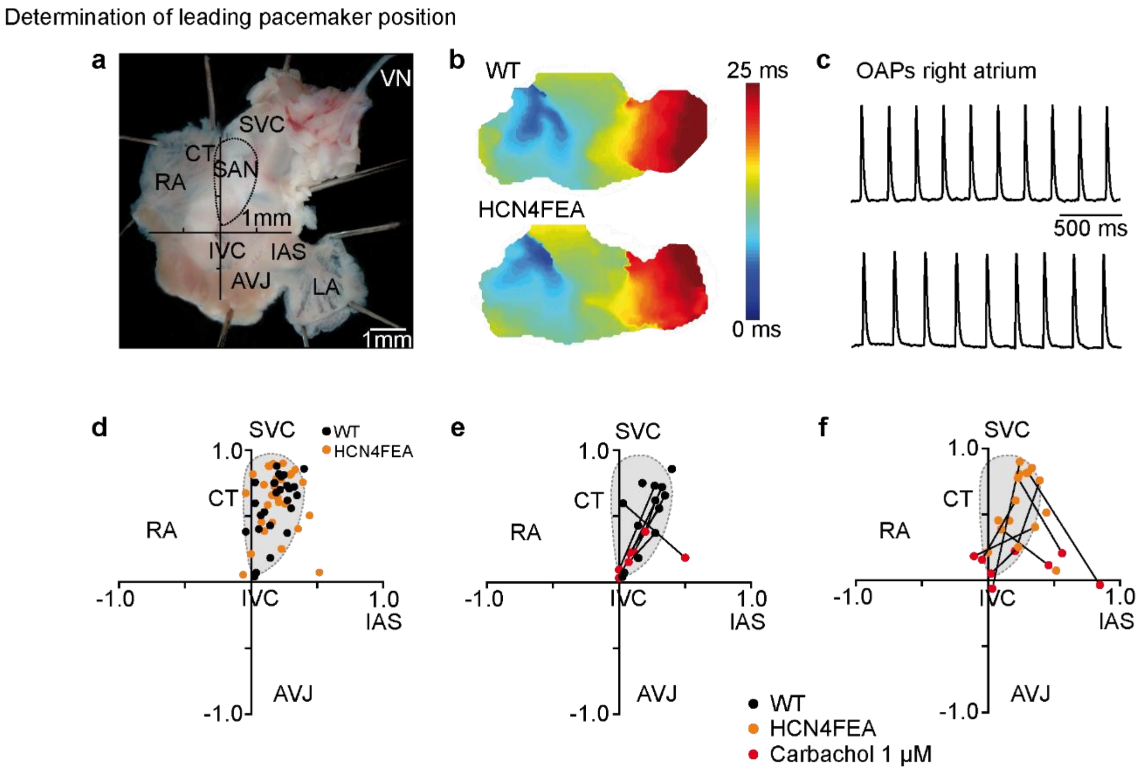

Supplementary Figure 2: Leading pacemaker site before and after application of a parasympathomimetic drug. a, Anatomy of a biatrial preparation with intact vagal nerve used for optical imaging. RA: right atrium; SAN: sinoatrial node; CT: crista terminalis; IVC: inferior caval vein; SVC: superior caval vein; AVJ: atrioventricular junction; IAS: interatrial septum; LA: left atrium; VN: vagal nerve. Orthogonal axes crossing at the IVC were used to plot the location of the leading pacemaker site and to quantify the magnitude of the leading pacemaker shift (n = 26 WT + 31 HCN4FEA biologically independent samples). **b,** Representative biatrial activation maps and **c,** corresponding

OAPs of WT and HCN4FEA preparations during spontaneous rhythm. **d,** Position of the leading pacemaker site within the SAN territory for each WT (n = 26) and HCN4FEA (n = 31) preparation. **e,** Leading pacemaker position before (black dots) and during application of carbachol (1 µM; red dots) in WT (n = 13). **f,** Leading pacemaker position before (orange dots) and during application of carbachol (1 µM; red dots) in HCN4FEA (n = 16). Shifts were larger in SANs of HCN4FEA mice. Female mice were used for optical imaging experiments. Source data are provided as a Source Data file.

Supplementary Figure 3

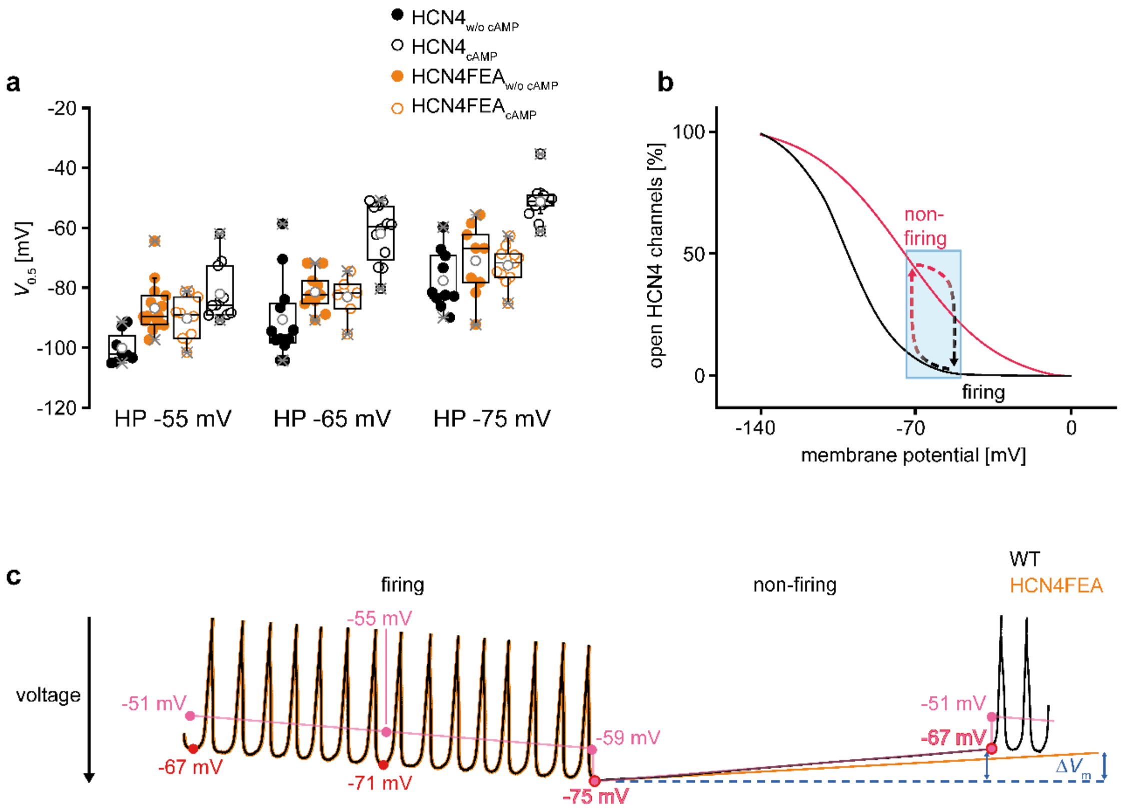

Supplementary Figure 3: Transition of the voltage dependence of HCN4 during firing and non-firing. a, $V_{0.5}$ values obtained from measurements of HCN4 currents in stably transfected HEK293 cells using different holding potentials. More negative holding potentials shift $V_{0.5}$ values to more positive potentials. Values from HCN4FEA mutant channels lie in between values determined from wild-type HCN4 channels without and with cAMP (HP -55 mV: HCN4 w/o cAMP n=8 cells, HCN4 cAMP n=11 cells, HCN4FEA w/o cAMP n=15 cells, HCN4FEA cAMP n=10 cells; HP -65 mV: HCN4 w/o cAMP n=12 cells, HCN4 cAMP n=12 cells, HCN4FEA w/o cAMP n=10 cells, HCN4FEA cAMP n=7 cells; HP -75 mV: HCN4 w/o cAMP n=10 cells, HCN4 cAMP n=13 cells, HCN4FEA w/o cAMP n=9 cells, HCN4FEA cAMP n=11 cells). **b,** Qualitative model to describe the transition of voltage dependence of HCN4 in SAN cells during firing and non-firing. **c,** Qualitative model for a typical cycle of firing and non-firing in SAN pacemaker cells. Firing starts at a MDP of -67 mV which slowly declines to -75 mV. At the same time, the average membrane potential declines from -51 mV (beginning of firing; pink curve) to -59 mV (end of firing; pink curve). When firing terminates, membrane potential abruptly drops to -75 mV (pink curve). During non-firing the membrane potential slowly rises to -67 mV until firing is reinitiated which leads to an abrupt increase in average potential to -51 mV. We propose that during firing and non-firing pronounced shifts in the activation curves of HCN4 channels **(b)** occur, which are driven by the large voltage steps at the beginning and the end of firing (**c**, step-like jumps in the pink curve). We suggest that by the end of the firing mode (-59 mV) the activation curve of HCN4 is positioned to more hyperpolarised potentials (**b**, black curve). After the abrupt jump to -75 mV at the beginning of non-firing the activation curve shifts towards more depolarised potentials (**b**, red curve). Once the threshold for firing is reached pacemaker cells switch to firing mode and voltage abruptly jumps to -51 mV, shifting voltage dependent activation to more hyperpolarised potentials (**b**, black curve) and the cycle repeats. Note that for the physiological model (**c**) all values are corrected for liquid junction potential. Boxplots show the median line, perc 25/75, and min/max value; grey open symbols represent the mean value. Source data are provided as a Source Data file.

Supplementary Figure 4

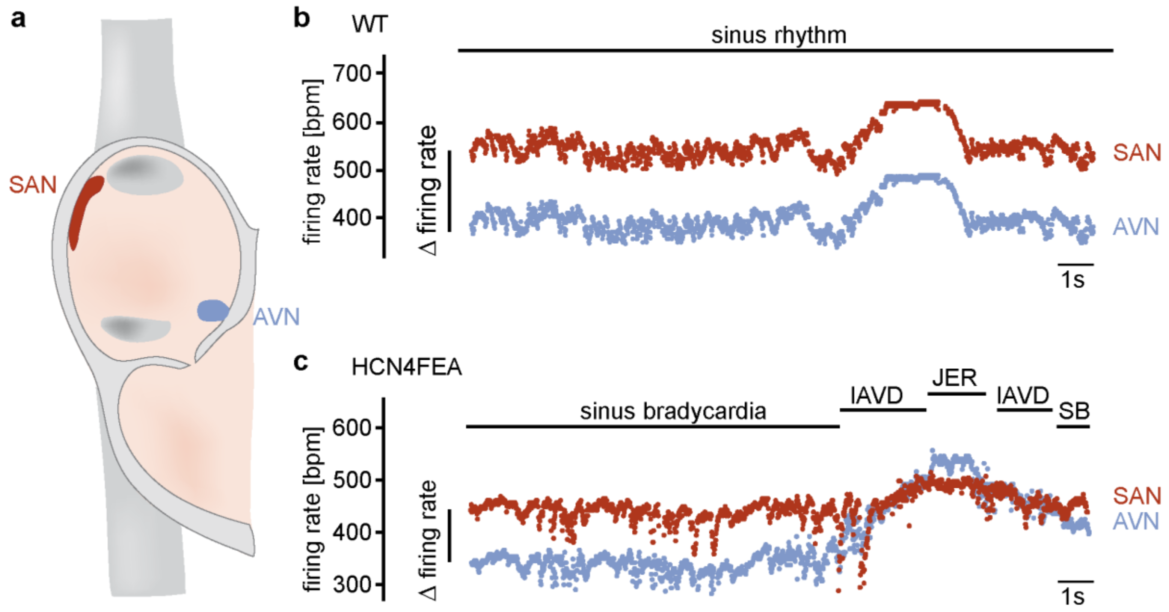

Supplementary Figure 4: Bradycardia and chronotropic incompetence disrupt the concerted action of SAN and AVN. a, Scheme depicting the positioning of SAN and AVN within the right atrium of the heart. **b,** SAN firing rate and putative AVN firing rate in a WT heart during regular sinus rhythm, HR acceleration, high HRs, and HR deceleration. SAN firing rate is always higher than the AVN firing rate and suppresses AVN activity. **c,** Sinus bradycardia (SB) caused by lack of CDR in HCN4FEA positions the SAN firing rate closer to that of the AVN. The combination of chronotropic incompetence of the SAN and preserved chronotropic competence of the AVN leads to escape phenomena (IAVD and JER) during sympathetic stimulation (see Discussion for details).
