# Supplementary material for: cAMP-dependent regulation of HCN4 controls the tonic entrainment process in sinoatrial node pacemaker cells
Source: Nat Commun. 2020 Nov 3;11:5555. doi: 10.1038/s41467-020-19304-9 (PMC7641277; doi:10.1038/s41467-020-19304-9)
Supplement: Supplementary file 2 — Description of Additional Supplementary Files [file 41467_2020_19304_MOESM2_ESM.docx]

File Name: Supplementary Data 1

Description: Voltage clamp data. Parameters with mean values +/- SEM and n numbers are given in row 3-29. Results of statistical analysis are given in row 32-98. Column B: parameter, Column C-D: statistical test method, Column E-H: groups being compared, Column I-J: p-values, Column K-L: significance level. For all statistical tests p < 0.05 was considered significant (***p < 0.001, **p < 0.005, *p < 0.05, ns = not statistically significant p > 0.05). Abbreviations: cAMP: cyclic adenosine monophosphate, HCN: hyperpolarisation-activated cyclic nucleotide-gated channel, SAN: sinoatrial node, WT: wild type, t: time constant of single or double exponential fits to current traces, k: slope factor of activation curves, CD: current density, V0.5: half-maximal activation voltage of HCN channels. Terms: [parameter]_w/o cAMP_: no cAMP was added to the intracellular solution when measuring in whole cell mode, [parameter]_cAMP_: cAMP was added to the intracellular solution when measuring in whole cell mode, WT: control group.

File Name: Supplementary Data 2

Description: Parameters with mean values +/- SEM, n numbers and results of statistical analysis are given in row 3-44. For all statistical tests p < 0.05 was considered significant (***p < 0.001, **p < 0.005, *p < 0.05, ns= not statistically, significant p > 0.05). Abbreviations: HCN: hyperpolarisation-activated cyclic nucleotide-gated channel, LV: left ventricle, qRT-PCR: quantitative real-time polymerase chain reaction, SAN: sinoatrial node, WT: wild type. Terms: ALAS: Gene encoding for aminolevulinic acid synthase: housekeeping gene used for normalization of data, HCN4FEA: test group expressing cAMP insensitive HCN4 channel protein, [parameter]_d_: parameter during diastole, [parameter]_s_: parameter during systole, WT: control group. Parameters: CO: cardiac output, EF: ejection fraction, FS: fractional shortening, HR: heart rate, IVS: interventricular septum thickness, LVID: LV internal dimension, LVPW: LV posterior wall thickness, SV: stroke volume.

File Name: Supplementary Data 3

Description: mRNA microarray data. Two-sided Welch´s t-test. Fold-change 1.5 with a p-value <0.05 was considered significant. Note: p values were not corrected for multiple testing. Abbreviations: HCN hyperpolarisation-activated cyclic nucleotide-gated channel. Terms: HCN4FEA: test group expressing cAMP insensitive HCN4 channel protein, WT: control group.

File Name: Supplementary Data 4

Description: Telemetric ECG data. Parameters with mean values +/- SEM, n numbers and results of statistical analysis are given in row 3-64. For all statistical tests p < 0.05 was considered significant (***p < 0.001, **p < 0.005, *p < 0.05, ns= not statistically significant p > 0.05). Abbreviations: ECG: electrocardiogram, HA: high activity, HCN: hyperpolarisation-activated cyclic nucleotide-gated channel, HF: high frequency, HRV: heart rate variability, LA: low activity, LF: low frequency, reg: regulation, VLF: very low frequency, WT: wild type. Terms: HCN4FEA: test group expressing cAMP insensitive HCN4 channel protein, [parameter]_basal_: parameter without any external drug administration, [parameter]_P_: parameter after propranolol injection, [parameter]_P+A_: parameter after propranolol and atropine injection, WT: control group. Parameters: HR: heart rate, IAVD: isorhythmic atrioventricular dissociation, JER: junctional escape rhythm, RMSSD: root mean square of successive differences, SDNN: standard deviation of N to N intervals.

File Name: Supplementary Data 5

Description: *In vivo* EPS data. Parameters with mean values +/- SEM, n numbers and results of statistical analysis are given in row 3-83. For all statistical tests p < 0.05 was considered significant (***p < 0.001, **p < 0.005, *p < 0.05, ns= not statistically significant p > 0.05). Abbreviations: EPS: electrophysiological study, HCN: hyperpolarisation-activated cyclic nucleotide-gated channel, WT: wild type. Terms: HCN4FEA: test group expressing cAMP insensitive HCN4 channel protein, [parameter]_80_: parameter at a cycle length of 80 ms, [parameter]_90_: parameter at a cycle length of 90 ms, [parameter]_100_: parameter at a cycle length of 100 ms, WT: control group. Parameters: AERP: right atrial effective refractory period, AVERP: atrioventricular node effective refractory period, AVFRP: atrioventricular node functional refractory period, AVNERP: atrioventricular nodal refractory period, AVRRP: atrioventricular node relative refractory period, SACT: sinoatrial conduction time, cSNRT: corrected sinus node recovery time, SNRT: sinus node recovery time, VERP: ventricular effective refractory period, WBP: Wenckebach periodicity.

File Name: Supplementary Data 6

Description: Wholemount SAN data. Parameters with mean values +/- SEM, n numbers and results of statistical analysis are given in row 3-36. For all statistical tests p < 0.05 was considered significant (***p < 0.001, **p < 0.005, *p < 0.05, ns= not statistically significant p > 0.05). Abbreviations: HCN: hyperpolarisation-activated cyclic nucleotide-gated channel, LP: leading pacemaker, SAN: sinoatrial node, WT: wild type. Terms: HCN4FEA: test group expressing cAMP insensitive HCN4 channel protein, [parameter]_basal_: parameter without external stimulation, [parameter]_Carbachol_: parameter after application of carbachol, [parameter]_20Hz_: parameter at a vagal nerve stimulation frequency of 20 Hz, TAT-TRIP8bnano: cell-penetrating peptide that antagonises cAMP binding to HCN channels, WT: control group. Parameters: RMSSD: root mean square of successive differences, SACT: sinoatrial conduction time, SDNN: standard deviation of N to N intervals.

File Name: Supplementary Data 7

Description: Patch clamp data of SAN cells. Parameters with mean values +/- SEM and n numbers are given in row 3-53. Results of statistical analyses are given in row 56-124. Column B: parameter, Column C-D: statistical test method, Column E-H: groups being compared, Column I-J: p-values, Column K-L: significance level. For all statistical tests p < 0.05 was considered significant (***p < 0.001, **p < 0.005, *p < 0.05, ns= not statistically significant p > 0.05). Abbreviations: HCN: hyperpolarisation-activated cyclic nucleotide-gated channel, I_f_: funny current, I_Ca, L_: L-type calcium channel current, I_Ca, T_: T-type calcium channel current, SAN: sinoatrial node, WT: wild type. Terms: HCN4FEA: test group expressing cAMP insensitive HCN4 channel protein, [parameter]_basal_: parameter without drug application, [parameter]_before Iso_: parameter without application of isoproterenol, [parameter]_Iso_: parameter with application of 100 nM isoproterenol, [parameter]_10nM_: parameter with application of 10 nM carbachol on the extracellular side, [parameter]_100nM_: parameter with application of 100 nM carbachol on the extracellular side, [parameter]_1000nM_: parameter with application of 1000 nM carbachol on the extracellular side, [parameter]_w/o cAMP_: no cAMP was added to the intracellular solution when measuring in whole cell mode, [parameter]_cAMP_: cAMP was added to the intracellular solution when measuring in whole cell mode, [parameter]_episodic firing_: parameter for cells that show episodic firing, [parameter]_permanent firing_: parameter for cells that show permanent firing, WT: control group. Parameters: C slow: cell capacitance, MDP: maximum diastolic potential, SDD: slow diastolic depolarisation, ΔVm: difference in hyperpolarised membrane potential between firing and non-firing mode, Reduction: decline in firing rate due to application of carbachol.

File Name: Supplementary Data 8

Description: Langendorff-perfused hearts. Parameters with mean values +/- SEM, n numbers and results of statistical analysis are given in row 3-27. For all statistical tests p < 0.05 was considered significant (***p < 0.001, **p < 0.005, *p < 0.05, ns= not statistically significant p > 0.05). Abbreviations: HCN: hyperpolarisation-activated cyclic nucleotide-gated channel, WT: wild type. Terms: HCN4FEA: test group expressing cAMP insensitive HCN4 channel protein, [parameter]_3Hz_: parameter at a vagal nerve stimulation frequency of 3 Hz, [parameter]_5Hz_: parameter at a vagal nerve stimulation frequency of 5 Hz, [parameter]_10Hz_: parameter at a vagal nerve stimulation frequency of 10 Hz, [parameter]_20Hz_: parameter at a vagal nerve stimulation frequency of 20 Hz, [parameter]_30Hz_: parameter at a vagal nerve stimulation frequency of 30 Hz, WT: control group. Parameters: HR: heart rate, Mean length: mean length of a sinus pause, RMSSD: root mean square of successive differences, SDNN: standard deviation of N to N intervals.

File Name: Supplementary Data 9

Description: Combined telemetric ECG and blood pressure data. Parameters with mean values +/- SEM, n numbers and results of statistical analysis are given in row 3-10. For all statistical tests p < 0.05 was considered significant (***p < 0.001, **p < 0.005, *p < 0.05, ns= not statistically significant p > 0.05). Abbreviations: BP: blood pressure, ECG: electrocardiogram, HCN: hyperpolarisation-activated cyclic nucleotide-gated channel, WT: wild type. Terms: HCN4FEA: test group expressing cAMP insensitive HCN4 channel protein, [parameter]_up_: parameter for up-sequences, [parameter]_down_: parameter for down-sequences WT: control group. Parameters: DBP: diastolic blood pressure, SBP: systolic blood pressure.

File Name: Supplementary Movie 1

Description: 2D laser scanning confocal microscopy movie of intracellular calcium fluorescence signals. Rhythmic and global calcium signals recorded in a WT whole-mount SAN preparation.

File Name: Supplementary Movie 2

Description: Ca^2+^ waves spreading bidirectionally along the long axis recorded in an HCN4FEA whole-mount SAN preparation.

File Name: Supplementary Movie 3

Description: Ca^2+^ waves spreading bidirectionally along the long axis recorded in a WT whole-mount SAN preparation after application of TAT-TRIP8bnano.

File Name: Supplementary Movie 4

Description: Spontaneous and highly localised Ca^2+^ events during diastole and global Ca transients during systole recorded in an HCN4FEA whole-mount SAN preparation.

File Name: Supplementary Movie 5

Description: Ca^2+^ waves propagating from one end of the cell to the other recorded in an HCN4FEA whole-mount SAN preparation.

File Name: Supplementary Movie 6

Description: Central Ca^2+^ waves spreading bidirectionally along the long axis recorded in an HCN4FEA whole-mount SAN preparation.
