# Supplementary material for: cAMP-dependent regulation of HCN4 controls the tonic entrainment process in sinoatrial node pacemaker cells
Source: Nat Commun. 2020 Nov 3;11:5555. doi: 10.1038/s41467-020-19304-9 (PMC7641277; doi:10.1038/s41467-020-19304-9)
Supplement: Supplementary file 18 — Reporting Summary [file 41467_2020_19304_MOESM18_ESM.pdf]

nature research

Corresponding author(s): Christian Wahl-Schott

Last updated by author(s): Sep 10, 2020

Reporting Summary

Nature Research wishes to improve the reproducibility of the work that we publish. This form provides structure for consistency and transparency in reporting. For further information on Nature Research policies, see Authors & Referees and the Editorial Policy Checklist .

Statistics

For all statistical analyses, confirm that the following items are present in the figure legend, table legend, main text, or Methods section.

n/a	Confirmed	
☐	☒	The exact sample size (*n*) for each experimental group/condition, given as a discrete number and unit of measurement
☐	☒	A statement on whether measurements were taken from distinct samples or whether the same sample was measured repeatedly
☐	☒	The statistical test(s) used AND whether they are one- or two-sided<br>*Only common tests should be described solely by name; describe more complex techniques in the Methods section.*
☐	☒	A description of all covariates tested
☐	☒	A description of any assumptions or corrections, such as tests of normality and adjustment for multiple comparisons
☐	☒	A full description of the statistical parameters including central tendency (e.g. means) or other basic estimates (e.g. regression coefficient) AND variation (e.g. standard deviation) or associated estimates of uncertainty (e.g. confidence intervals)
☐	☒	For null hypothesis testing, the test statistic (e.g. *F*, *t*, *r*) with confidence intervals, effect sizes, degrees of freedom and *P* value noted<br>*Give P values as exact values whenever suitable.*
☒	☐	For Bayesian analysis, information on the choice of priors and Markov chain Monte Carlo settings
☒	☐	For hierarchical and complex designs, identification of the appropriate level for tests and full reporting of outcomes
☒	☐	Estimates of effect sizes (e.g. Cohen's *d*, Pearson's *r*), indicating how they were calculated

Our web collection on statistics for biologists contains articles on many of the points above.

Software and code

Policy information about availability of computer code

Data collection	Optical Imaging: BV_Ana Ver.1604, Brainvision Inc.; MiCAM05 data acquisition software Ver. 2.5.4, Brainvision Inc.; LabChart 8, ADInstruments<br>ECG and blood pressure telemetry: Dataquest ART 3.1, Data Science International<br>In vitro electrophysiology: Clampex 10.5.2.6, Molecular Devices, LLC; PatchMaster v2x90.2, HEKA Elektronik Dr. Schulze GmbH<br>In vivo electrophysiology: EP-Tracer_V1.05, Schwarzer Cardiotek<br>Langendorff heart and in vitro ECG: LabChart 8, ADInstruments<br>Confocal microscopy and imaging: Las X, Leica Microsystems<br>Microscopy: ZenCore 5.3, Carl Zeiss AG; cellSens 2.3, Olympus
Data analysis	In vitro electrophysiology: Clampfit 10.5.2.6, Molecular Devices; FitMaster v2x90.2, HEKA Elektronik Dr. Schulze GmbH<br>ECG and blood pressure telemetry: ecgAUTO v3.3.5.10, EMKA Technologies<br>In vivo electrophysiology: EP-Tracer_V1.05, Schwarzer Cardiotek<br>Microarray: Expression Console 1.0, Affymetrix; Partek Genomics Suite 6.5, Partek<br>SAN cell size, fibrosis: ImageJ 1.50i<br>Optical Imaging: MATLAB R2015b, MathWorks; BV_Ana Ver.1604, Brainvision Inc.; Rhythm 2012 (MATLAB-based), Laughner et al., 2012, https://efimovlab.seas.gwu.edu/rhythm/; github.com/rrasheed/rhythm-analysis-software<br>Crystal structure: PyMOL™ 2.2.0, Schrodinger, LLC

For manuscripts utilizing custom algorithms or software that are central to the research but not yet described in published literature, software must be made available to editors/reviewers. We strongly encourage code deposition in a community repository (e.g. GitHub). See the Nature Research guidelines for submitting code & software for further information.

Data

Policy information about availability of data

All manuscripts must include a data availability statement. This statement should provide the following information, where applicable:

- Accession codes, unique identifiers, or web links for publicly available datasets
- A list of figures that have associated raw data
- A description of any restrictions on data availability

The Microarray dataset generated and analysed during the current study is available in the National Center for Biotechnology Information Gene Expression Omnibus (GEO) repository and is accessible through the GEO Series accession number GSE138086.

The source data underlying Figure 1e-f; Figure 2e-f, h, j-k; Figure 3c, f, j; Figure 4e-i; Figure 5c-e; Figure 6c; Figure 7d, f-g, j-k; Figure 8b; Figure 9c; Supplementary Figure 1a, c, g, i-k; Supplementary Figure 2d-f and Supplementary Figure 3a Fig. 1E-F, H, J-K; Fig. 2C, F, J; Fig. 3E-I; Fig. 4C; Fig. 5D, F-G, J-K; Fig. 6B; Fig. 7C and supplementary Fig. IID; Fig. IIIA, C, G, I-K; Fig. IVD, G-I, L-M are provided with this paper as Source Data file.

Any other datasets generated during and/or analysed during the current study are available from the corresponding author upon reasonable request.

The Figure of the C-terminus structure was prepared using PyMOL. The structure is based on sequence 521 - 723 of the human HCN4 structure in complex with cAMP, downloaded from the protein data base (accession code: 3U11).

Field-specific reporting

Please select the one below that is the best fit for your research. If you are not sure, read the appropriate sections before making your selection.

☒ Life sciences ☐ Behavioural & social sciences ☐ Ecological, evolutionary & environmental sciences

For a reference copy of the document with all sections, see nature.com/documents/nr-reporting-summary-flat.pdf

Life sciences study design

All studies must disclose on these points even when the disclosure is negative.

Sample size	No statistical methods were used to predetermine sample size. Sample sizes were chosen based on previous experience and considered to be sufficient based on publications in the field (Fenske et al [1, 20, 38]; Mesirca et al [21, 43]; Kozasa et al [22]; Manniko et al [29]; Laude et al [34, 35]; Glukhov et al [47]; Efimov et al [51]<br>At least triplicates were used to meet the minimal requirements for statistical analysis.<br>Effort was taken to keep the number of animals at a minimum.
Data exclusions	Animals with unsufficient quality of the ECG and/or blood pressure signal were excluded from the study.<br>Possible technical problems during in-vivo EPS that lead to data exclusion were measurements in which the stimulus did not always lead to a capture/response, in which an animal's spontaneous heart rate was temporarily or permanently faster than the S1S1 stimulus interval, or premature termination of the experiment due to an uncontrollable stage of anaesthesia.<br>All exclusion criteria were pre-established
Replication	All animal studies and in vitro electrophysiology with mouse tissue were performed with littermates from multiple litters over time.<br>Histology and immunohistology were performed at least three times independently.<br>Western blot analysis from SAN tissue and microarray was performed once.<br>All attempts at replication were successful.
Randomization	All animals and cells were randomly assigned to the experimental group.
Blinding	In vivo telemetry data analysis was not blinded because the ECG phenotype of the HCN4FEA animals was obvious.<br>Analysis of all other in vivo experiments was blinded.<br>Microscopy data analysis was performed in a blinded way, as this method is very sensitive to experimenter's biases but sample preparation, histological and biochemical experiments were not performed in a blinded way.<br>qPCR values were normalized to the internal control, thus blinded experiment is not necessary.<br>Data analysis of voltage-clamp electrophysiology was not blinded but performed independently by two researchers.<br>Data analysis of current-clamp electrophysiology was not blinded but in some cases performed independently by two researchers.<br>Optical imaging data analysis was mainly performed blinded but sample preparation and measurements were not blinded.<br>All other experiments were not performed or analysed in a blinded way.

Reporting for specific materials, systems and methods

We require information from authors about some types of materials, experimental systems and methods used in many studies. Here, indicate whether each material, system or method listed is relevant to your study. If you are not sure if a list item applies to your research, read the appropriate section before selecting a response.

Materials & experimental systems

n/a	Involved in the study
☐	☒ Antibodies
☐	☒ Eukaryotic cell lines
☒	☐ Palaeontology
☐	☒ Animals and other organisms
☒	☐ Human research participants
☒	☐ Clinical data

Methods

n/a	Involved in the study
☐	☒ ChIP-seq
☒	☐ Flow cytometry
☒	☐ MRI-based neuroimaging

Antibodies

Antibodies used

Guinea pig polyclonal anti-HCN4, supplier: Alomone Labs, catalog number: AGP-004, polyclonal antibody, Lot AGP004AN0202
Validation: HCN4 expression was only detected in the sinoatrial node of explants and was absent in surrounding atrial tissue as shown in Supplemental Figure 1F,G. Additionally the antibody was validated by the manufacturer.

Rabbit polyclonal anti-HCN1, supplier: Alomone Labs, catalog number: APC-056, polyclonal antibody, Lot APC056AN1402
Validation: HCN1 expression was only detected in the sinoatrial node of explants and was absent in surrounding atrial tissue as shown in Supplemental Figure 1F,G. Additionally the antibody was validated by the manufacturer.

Mouse anti-HCN1, supplier: Abcam, catalog number: ab176304, polyclonal antibody, Lot n/a.
Validation: The antibody was validated by the manufacturer by western blot analysis using lysates from murine tissues and several cell lines.

Rat anti-HCN4, supplier: Santa Cruz Biotechnology, catalog number: Sc-58622, clone SHG 1E5, Lot n/a.
The antibody was validated by the manufacturer by western blot analysis using whole cell lysates from several cell lines.

Rabbit anti-HCN2 L, supplier: Ludwig et al., 2003, catalog number: n/a, clone n/a, Lot n/a (commercially not availlable, availlable on request from the authors).
Validation: Ludwig et al., 2003 confirmed presence of HCN2 in western blot analysis of WT brains and absence of the protein in HCN2 knock-out animals, Figure 1D

Mouse anti-beta-tubulin, supplier: Developmental Studies Hybridoma Bank (DSHB), catalog number: E7, clone E7, Lot n/a.

Cy3 donkey anti-rabbit IgG, supplier: Merck Millipore, catalog number: AP182C, polyclonal antibody, Lot 2912152

FITC donkey anti-guinea pig IgG, supplier: Merck Millipore, catalog number: AP193F, polyclonal antibody, Lot 2929222

Validation

please see above

Eukaryotic cell lines

Policy information about cell lines

Cell line source(s)

HEK293 cells were purchased from ATCC.
Flip-In™-293 cells were purchased from Thermo Fisher Scientific (catalog number: R75007).
Stable Flip-In™-293 HCN4WT and Flip-In™-293 HCN4FEA cell lines were generated in-house using the Flp-In Complete System (Invitrogen, catalog number: K601001).

Authentication

HEK293: ATCC states that they comprehensively perform authentication (Short Tandem Repeat profiling) and quality-control tests on all distribution lots of cell lines. In cell culture the HEK cells had shown typical morphology and cell growth.
Flip-In™-293: cell lines were not authenticated

Mycoplasma contamination

Cell lines were not tested for mycoplasma contamination.

Commonly misidentified lines
(See ICLAC register)

No commonly misidentified cell lines were used.

Animals and other organisms

Policy information about studies involving animals; ARRIVE guidelines recommended for reporting animal research

Laboratory animals	WT and HCN4FEA (Hcn4tm3(Y527F;R669E;T670A)Biel) mice were obtained by in-house breeding and maintained on a mixed C57BL/6N and 129/SvJ background.<br><br>All in vivo measurements were carried out in male animals because there are significant sex differences in heart rate and arterial blood pressure. Some of the in vitro data were performed using female animals to reduce the overall number of animals in the study (according to the 3R rules by Russel and Burch). We define in the detailed methods section in the online supplement which experiments were performed in male and female mice, respectively. Sexes were never mixed in a single experiment.<br><br>Age of animals used in the experiments:<br>ECG telemetry: 5-6 months<br>Combined ECG and blood presure telemetry: 4 months<br>In vivo electrophysiology: 2-3 months<br>In vitro electrophysiology, SAN cells: 10 weeks<br>In vitro electrophysiology, isolated hearts: 8-10 weeks<br>Optical imaging: 3 months<br>SAN histology, HE staining, Sirius Red Fast Green staining, Western Blot and confocal calcium imaging: 11-13 weeks
Wild animals	No wild animals were used.
Field-collected samples	No field-collected samples were used.
Ethics oversight	All animal studies were approved by the Regierung von Oberbayern, were in accordance with German laws on animal experimentation, and were performed in compliance with widely accepted ethical standards. Effort was taken to keep the number of animals at a minimum.

Note that full information on the approval of the study protocol must also be provided in the manuscript.

ChIP-seq

Data deposition

- [x] Confirm that both raw and final processed data have been deposited in a public database such as GEO.
- [ ] Confirm that you have deposited or provided access to graph files (e.g. BED files) for the called peaks.

Data access links<br>*May remain private before publication.*	data accessible at NCBI GEO database, accession GSE138086
Files in database submission	1 Excel file containing metadata worksheet and processed data in matrix table format<br>6 CEL-files
Genome browser session<br>(e.g. UCSC)	*Provide a link to an anonymized genome browser session for "Initial submission" and "Revised version" documents only, to enable peer review. Write "no longer applicable" for "Final submission" documents.*

Methodology

Replicates	1
Sequencing depth	n/a
Antibodies	n/a
Peak calling parameters	n/a
Data quality	n/a
Software	Quality of RNA specimen was checked on an Agilent BioAnalyzer 2100 (Agilent Technologies, Germany) and processed for Affymetrix Gene Chips using Affymetrix whole transcript sense target labeling kit (Affymetrix, USA). Fragmented and labeled cDNA was hybridized onto murine MouseGene1.1-ST Gene Chips. Staining of biotinylated cDNA and scanning of arrays were performed according to the manufacturer's recommendations. Raw CEL-files were imported into Expression Console 1.0. RMA (Affymetrix, USA) was used for array normalization and signal calculation. Normalized signal values were imported into Partek Genomics Suite 6.5.
